# Hydrostatic pressure induces strong leakage of dissolved organic matter from “marine snow” particles

**DOI:** 10.1126/sciadv.aec5677

**Published:** 2026-02-04

**Authors:** Peter Stief, Jutta Niggemann, Margot Bligh, Hagen Buck-Wiese, Urban Wünsch, Michael Steinke, Jan-Hendrik Hehemann, Ronnie N. Glud

**Affiliations:** ^1^ HADAL & Nordcee, Department of Biology, University of Southern Denmark, Odense, Denmark.; ^2^ ICBM-MPI Bridging Group for Marine Geochemistry, Institute for Chemistry and Biology of the Marine Environment, Carl von Ossietzky Universität Oldenburg, Oldenburg, Germany.; ^3^ MARUM-MPI Bridging Group for Marine Glycobiology, Center for Marine Environmental Sciences, University of Bremen, Bremen, Germany.; ^4^ Department of Marine and Environmental Biology, University of Southern California, Los Angeles, CA, USA.; ^5^ National Institute of Aquatic Resources, Section for Oceans and Arctic, Technical University of Denmark, Kongens Lyngby, Denmark.; ^6^ School of Life Sciences, University of Essex, Colchester, UK.; ^7^ Danish Institute for Advanced Study, University of Southern Denmark, Odense, Denmark.; ^8^ Tokyo University of Marine Science and Technology, Tokyo, Japan.

## Abstract

Marine snow forms at the ocean surface, sinks to depth, and ultimately enables carbon sequestration in the seabed. Fast-sinking marine snow particles, such as diatom aggregates, encounter a rapid increase in hydrostatic pressure during their descent. Using incubations in rotating pressure tanks, we found that pressure levels corresponding to 2- to 6-kilometer water depth induce leakage of dissolved organic matter (DOM) from diatom aggregates equivalent to ~50% of their initial carbon contents. The leaked DOM proved to be diatom-derived and changed the amount and composition of DOM in the surrounding seawater substantially. Ultrahigh-resolution mass spectrometry, high protein-like fluorescence, and low carbon:nitrogen ratios classified the leaked DOM as labile. The bioavailability of leaked DOM was demonstrated by its rapid utilization by a pelagic microbial community, leaving mainly recalcitrant DOM behind. Pressure-induced DOM leakage likely weakens the gravitational “biological carbon pump” and supplies labile DOM to the pelagic microbiome of the deep ocean.

## Teaser

Particles sinking into the deep ocean come under pressure and leak dissolved organic matter, which affects marine carbon cycling.

## INTRODUCTION

Particulate organic matter (POM) is the major form in which carbon fixed at the ocean surface is transported to the ocean interior. Gravitational sinking of organic particles drives the “biological carbon pump” (*1*–*3*), which sustains life in the ocean interior and facilitates sequestration of a small fraction of organic carbon in the seabed (*4*). During their descent through the water column, marine snow particles are degraded by heterotrophic microbes and grazing metazoans, which weakens the biological carbon pump (*5*, *6*). Osmotrophic microbes require POM to transition into dissolved organic matter (DOM) of sufficiently small molecular size to be taken up into their cells (*7*). DOM is generated from POM via four known pathways (*8*, *9*): (i) Living phytoplankton release fixed carbon as photosynthetic exudates and/or by-products of respiration (*10*, *11*), (ii) viral lysis of phytoplankton cells liberates intracellular solutes (*12*, *13*), (iii) metazoans grazing on phytoplankton release DOM through sloppy feeding (*14*), and (iv) exoenzymatic POM solubilization produces DOM that is available to both particle-associated and free-living microbes (*15*–*18*). Here, we introduce an additional pathway of DOM release from sinking POM, hydrostatic pressure–induced DOM leakage.

DOM release from sinking particles decreases the efficiency of the biological carbon pump and attenuates the vertical carbon export (*3*, *19*). Part of the released DOM is assimilated by ambient microbes, which thereby make it available to the pelagic food web, a process referred to as the “microbial loop” (*15*, *20*). Another part of the released DOM is mineralized by particle-attached and/or free-living microbes, which liberates CO_2_ that may be fixed again by photo- and chemotrophic primary producers. However, depending on the lability or recalcitrance of individual DOM constituents, microbial mineralization may take hours to millennia (*8*). Microbial mineralization of DOM (but also POM) produces a vast number of new DOM constituents that might end up in the persistent DOM pool of the ocean (*21*, *22*), which can be traced as characteristic fluorescence of recalcitrant DOM (*23*, *24*). This microbe-driven process of DOM diversification, referred to as the “microbial carbon pump,” has been proposed as a major carbon sequestration pathway in the water column (*8*, *25*, *26*).

The efficiency of the biological carbon pump critically depends on the depth at which the exported organic carbon is mineralized or lost as DOM (*27*–*29*). It is commonly assumed that particle-associated organic carbon arriving at depths of >1000 m remains out of contact with the atmosphere for >100 years, even if it is being mineralized to CO_2_ (*27*, *28*). Regionally, this “sequestration depth” might be as deep as 2000 m (*29*). Microbial particle degradation at these water depths will thus be affected by hydrostatic pressure. Experimental incubations have shown that high pressure significantly inhibits microbial particle degradation, likely because the surface-derived microbes colonizing the sinking particles are not adapted to high-pressure conditions (*30*–*34*). In addition, high pressure inhibits the activity of several particle-associated exoenzymes (*33*, *35*, *36*), which likely reduces enzymatic solubilization of POM to DOM.

When sediments are retrieved from the deep ocean, DOM leakage is observed (*37*, *38*). This artifact is explained by rapidly decreasing pressure levels, which may lyse the cells of benthic pro- and eukaryotes. Here, we explored the possibility of DOM leakage induced by increasing pressure levels as encountered by marine snow particles during their descent. Using rotating pressure tanks (*32*), we exposed model diatom aggregates and diatom cultures to slowly increasing pressure levels and analyzed the amount and composition of DOM both in the seawater and the diatom cells. We observed pressure-induced DOM leakage from diatoms at the expense of intracellular DOM. We then tested the bioavailability of the leaked DOM for natural seawater microbial communities to assess the implications of pressure-induced DOM leakage for the gravitational biological carbon pump and the pelagic microbiome of the deep ocean.

## RESULTS

### DOM leakage experiments

#### Experimental simulation

**Fig. 1.**
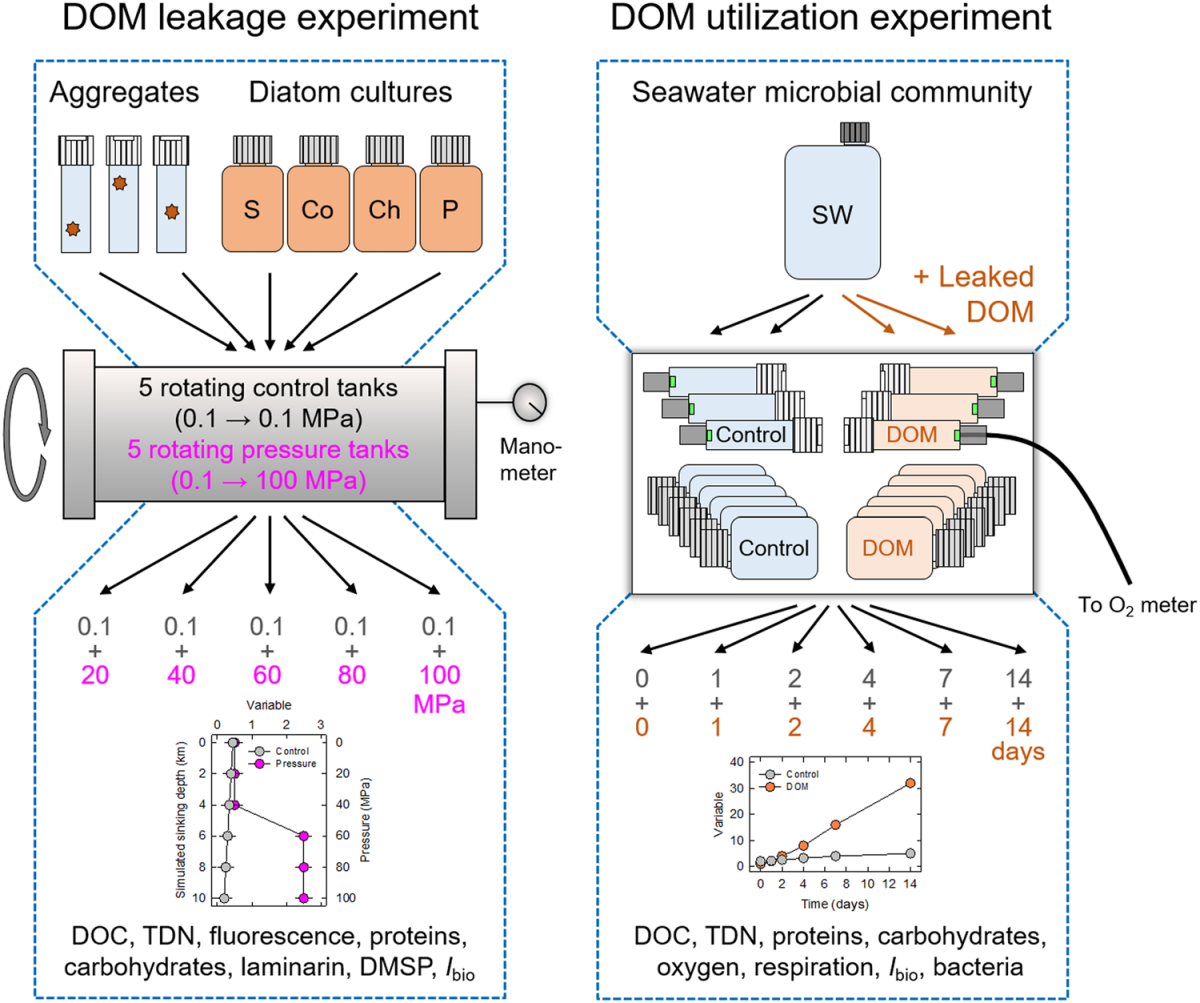
Experimental design. (**Left**) DOM leakage experiment with diatom aggregates and diatom cultures. S, *S. marinoi*; Co, *C. weissflogii*; Ch, *C. socialis*; P, *P. tricornutum*. Aggregates were individually incubated in 6-ml glass exetainers, while cultures were incubated in 30-ml polyethylene (PE) flasks. Samples were incubated in darkness and at 3°C in rotating incubation tanks that served as either pressure or control tanks. In the pressure tanks, hydrostatic pressure was increased by 5 MPa/day, thereby simulating the 20-day descent of diatoms from the surface ocean (0 km ≙ 0.1 MPa) into a deep-sea trench (10 km ≙ 100 MPa) (pressure treatment). In the control tanks, pressure was kept at atmospheric level (control treatment). Samples were retrieved from one pressure and one control tank each when 20, 40, 60, 80, and 100 MPa had been reached in the pressure tanks. This incubation and sampling scheme allowed reconstruction of depth profiles of pressure effects on different variables at 2-km resolution (see example plot). (**Right**) DOM utilization experiment with a natural seawater (SW) microbial community amended (DOM-amended treatment) or not amended with leaked DOM (control treatment) from pressure-exposed *S. marinoi*. Seawater was incubated in 6-ml glass exetainers equipped with an oxygen-sensing optode and in 30-ml polyethylene flasks in darkness and at 15°C and atmospheric pressure for 2 weeks. Samples were taken at predefined time points. This incubation and sampling scheme allowed following the temporal changes of different variables (see example plot).

The descent of diatom aggregates from the surface ocean into the deep ocean was simulated in rotating incubation tanks that kept the aggregates in suspension (*32*) (Fig. 1). In half of the tanks, hydrostatic pressure was incrementally increased by 5 MPa/day to lastly reach 100 MPa (“pressure” treatment), while in the other half of the tanks, pressure was always kept at 0.1 MPa (“control” treatment). The daily pressure increase by 5 MPa (≙ 50 bar ≙ 500 m in depth) corresponds to a simulated sinking velocity of 500 m/day, which is at the high end of in situ sinking velocities of diatom aggregates. The incubation temperature was 3°C. At the start of the 20-day incubations, the diatom aggregates had a volume of 5.8 ± 1.8 mm^3^ (means ± SD, *n* = 227), an equivalent spherical diameter of 2.2 ± 1.5 mm, *n* = 227), a diatom abundance of 1.6 × 10^5^ ± 0.3 × 10^5^ cells/mm^3^ (*n* = 35), total carbon and nitrogen contents of 6.2 ± 1.3 and 0.9 ± 0.2 μg/mm^3^, respectively (*n* = 50), and molar C:N ratios of 8.3 ± 2.5 (*n* = 50).

#### DOM bulk composition and fluorescence

**Fig. 2.**
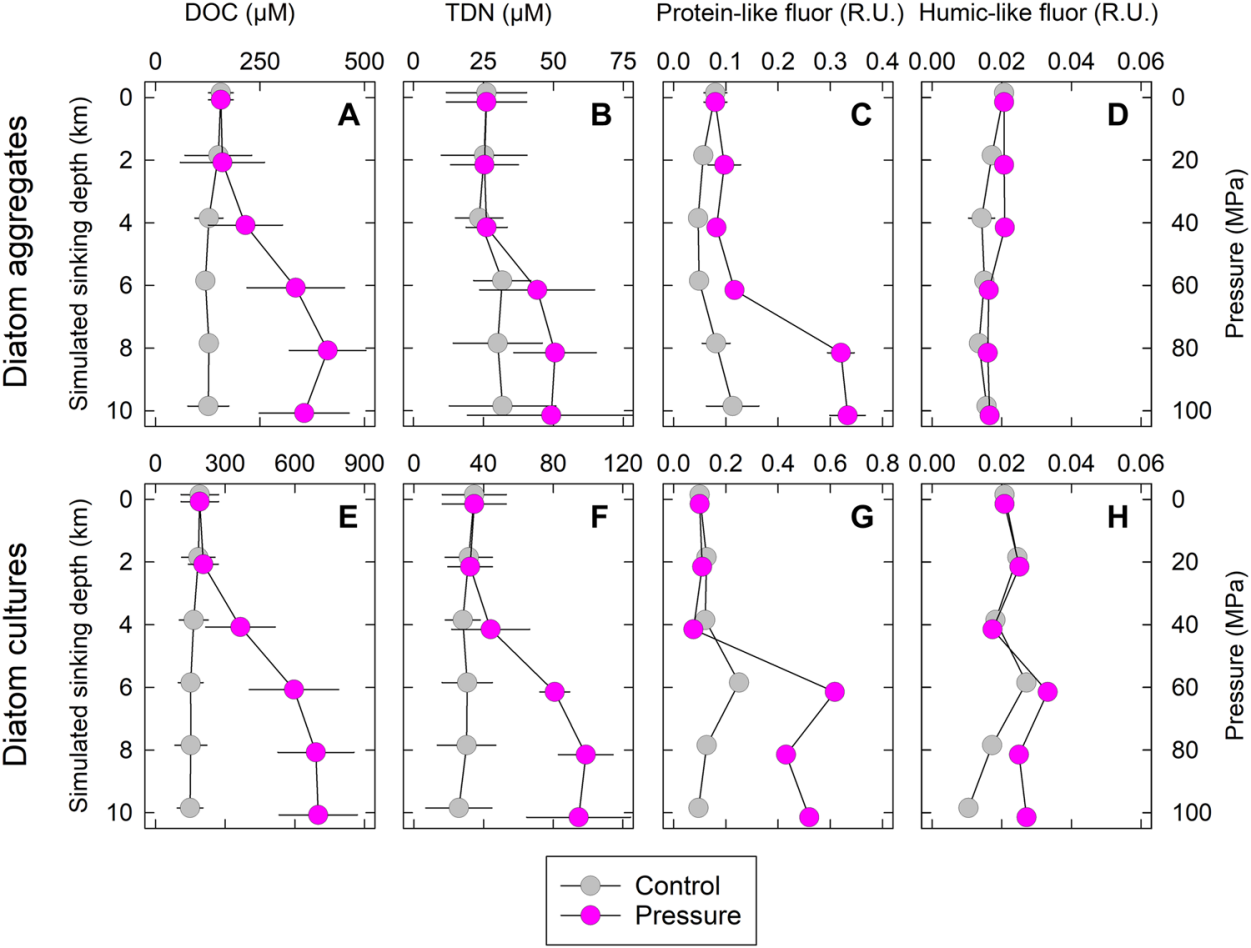
DOM leakage from diatom aggregates and diatom cultures. Bulk composition and fluorescence of DOM in incubations of (**A** to **D**) diatom aggregates and (**E** to **H**) cultures of the diatom *S. marinoi*. Hydrostatic pressure was increased by 5 MPa/day, thereby simulating the 20-day descent of aggregates or diatoms from the surface ocean (0 km ≙ 0.1 MPa) into a deep-sea trench (10 km ≙ 100 MPa) (pressure treatment). Pressure was kept at atmospheric level in parallel incubations (control treatment). Incubation temperature was 3°C. DOC, dissolved organic carbon; TDN, total dissolved nitrogen; Fluor, fluorescence; R.U., Raman units. Means ± SD of 21 aggregates incubated in five independent experiments and *S. marinoi* cultures incubated in four independent experiments are shown. For fluorescence, results from one aggregate experiment (with three replicate aggregates at each pressure level) and from one diatom experiment (with three technical replicates at each pressure level) are shown; error bars are sometimes smaller than the symbol.

Both the diatom aggregates and the diatom cultures from which they were produced (*Skeletonema marinoi*) showed net leakage of dissolved organic carbon (DOC) and total dissolved nitrogen (TDN) when 40 MPa was reached during the 20-day incubations (Fig. 2, A, B, E, and F). DOC and TDN concentrations in the surrounding water increased further when 60 MPa was reached and remained stable at pressures of >60 MPa. In the control incubation at atmospheric pressure, DOC and TDN concentrations remained low throughout the entire incubation. Protein-like DOM fluorescence in the surrounding water showed the same pressure-dependent patterns as DOC and TDN, while pressure-induced changes in humic-like fluorescence were less pronounced (Fig. 2, C, D, G, and H). The effect of pressure on extracellular DOC and TDN concentrations and on DOM fluorescence was statistically significant, except for humic-like DOM fluorescence (table S1).

#### DOM compound classes

**Fig. 3.**
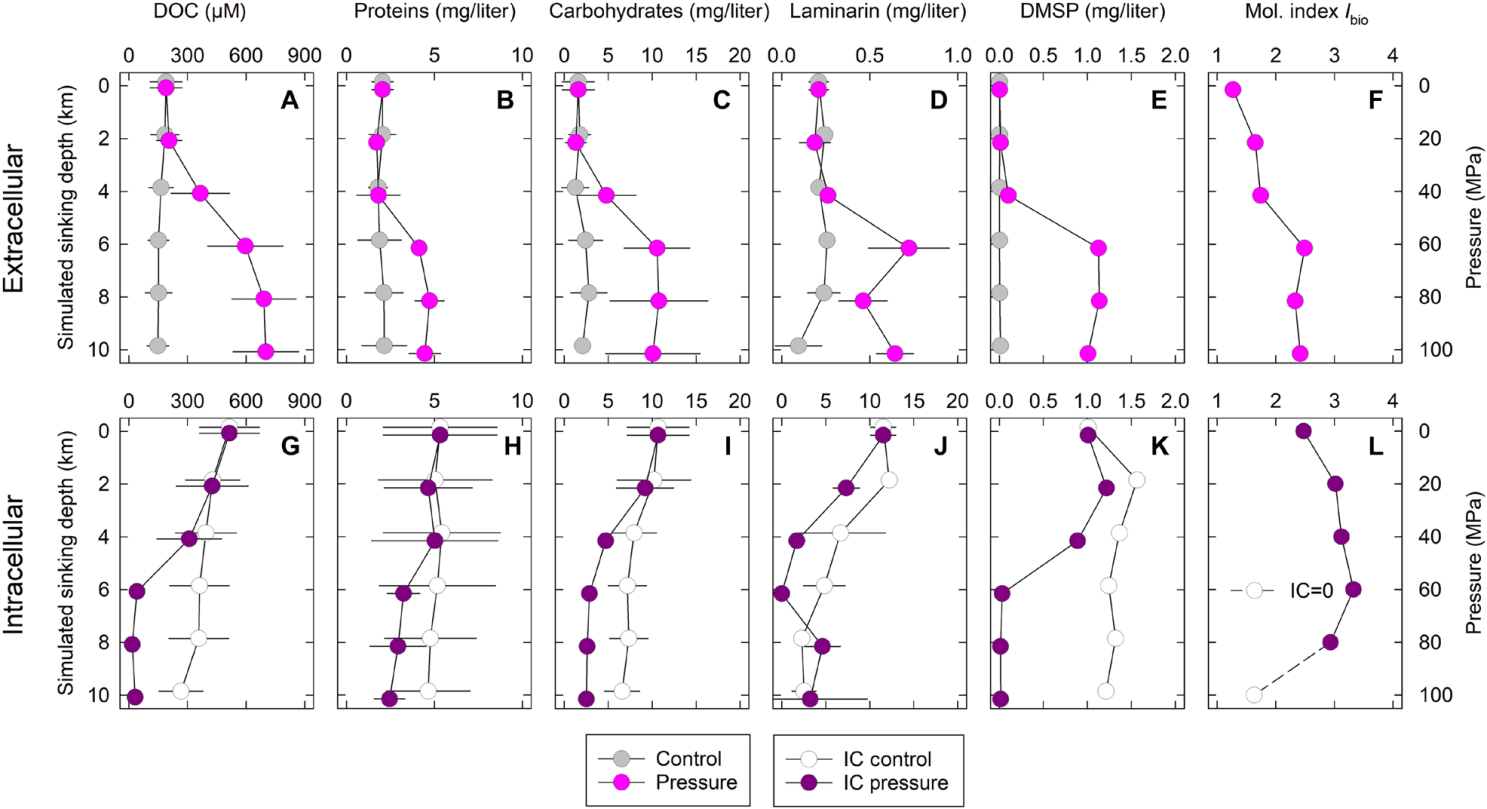
DOM leakage from diatom cultures. Composition of (**A** to **F**) extracellular and (**G** to **L**) intracellular DOM in incubations of *S. marinoi* cultures. Hydrostatic pressure was increased by 5 MPa/day, thereby simulating the 20-day descent of diatoms from the surface ocean (0 km ≙ 0.1 MPa) into a deep-sea trench (10 km ≙ 100 MPa) (pressure treatment). Pressure was kept at atmospheric level in parallel incubations (control treatment). Incubation temperature was 3°C. Extra- and intracellular concentrations are expressed per sample volume and can thus be directly compared. The unitless index for biological formation and transformation, *I*_bio_, was calculated from molecular DOM formulae obtained through Fourier transform ion cyclotron resonance mass spectrometry (FT-ICR-MS). High *I*_bio_ values suggest recent biological production of DOM of presumably high lability. Proteins, total proteins; carbohydrates, total hydrolysable carbohydrates. For DOC, proteins, and carbohydrates, means ± SD of *S. marinoi* cultures incubated in three to four independent experiments are shown; for laminarin, DMSP, and *I*_bio_, results from one pressure experiment are shown (here, error bars refer to technical replicates). Error bars are sometimes smaller than the symbol. Open symbol in (L) marks a questionable data point for *S. marinoi* cells depleted in intracellular DOM (IC = 0).

In *S. marinoi* incubations, extracellular concentrations of total proteins and carbohydrates, laminarin, and dimethylsulfoniopropionate (DMSP) generally showed the same pressure-dependent patterns as DOC and TDN (Fig. 3, A to E). Total carbohydrates were the most abundant compound class leaking from *S. marinoi*, with a small contribution (~5%) by the diatom-specific storage polysaccharide laminarin. Total proteins were the second most abundant compound class. DMSP, measured because of its potential role as a piezolyte in diatoms, was quantitatively unimportant but showed the most clear-cut concentration pattern.

Expressing the total protein and carbohydrate concentrations in carbon units allows constraining their relative contribution in the bulk DOC leachate of *S. marinoi*. At pressure levels of 60 to 100 MPa, bulk DOC leakage amounted to ~500 μM DOC and comprised ~100 μM proteins (assuming 53% C contents) and ~300 μM carbohydrates (assuming 44.5% C contents). This leaves ~100 μM for all other organic carbon compounds in the *S. marinoi* leachate.

#### Intracellular DOM

In the pressure treatment, increases in extracellular DOM concentrations were mirrored by decreases in intracellular DOM concentrations, both compound-(class)-specifically and quantitatively (Fig. 3, G to K). Hence, the observed pressure-induced DOM leakage occurred at the expense of intracellular DOM. Laminarin represented a notable exception to this rule, as its intracellular pool decreased irrespective of the pressure treatment (Fig. 3J). In the control treatment, much of the intracellular laminarin pool was obviously used by *S. marinoi*, as laminarin leakage did not occur. In the pressure treatment, the depletion of the intracellular laminarin pool was even stronger, but concomitant laminarin leakage was still minor. This implies that also under pressure much of the laminarin depletion was due to utilization by *S. marinoi* that survived the pressure treatment. The effect of pressure on extra- and intracellular DOC and TDN concentrations and on extracellular protein and carbohydrate concentrations was statistically significant (table S1).

Maximum DOM leakage from diatom aggregates corresponded to 50 and 58% of initial carbon and nitrogen contents, respectively (Table 1). Maximum DOM leakage from *S. marinoi* cells corresponded to 42 and 63% of the initial cellular carbon and nitrogen contents, respectively (Table 1). Multiplying per-cell DOC and TDN leakage by the cell abundance of *S. marinoi* in the aggregates results in DOC (2.6 μg of C/mm^3^) and TDN (0.4 μg of N/mm^3^) leakage from aggregates. This is quite consistent with the observed DOC and TDN leakage from aggregates of DOC (2.97 μg of C/mm^3^) and TDN (0.46 μg of N/mm^3^), respectively. Notably, the molar C:N ratio of the DOM leachates of diatom aggregates and *S. marinoi* cultures was lower than that of the aggregate and culture biomass used for experimentation (Table 1).

**Table 1.** Initial elemental and organic matter composition of diatom aggregates and *S. marinoi* cultures and maximum pressure-induced DOM leakage. Diatom aggregates and *S. marinoi* cultures were subject to 20-day pressure and control incubations (DOM leakage experiment). Subsamples for elemental and organic matter composition of aggregates and cultures were taken just before starting the incubations. Absolute DOM leakage per diatom aggregate volume (in micrograms per 1 mm^3^ of aggregate) and *S. marinoi* cell (in picograms per cell) was calculated as the difference in extracellular concentrations of the respective solute at elevated versus atmospheric pressure. Relative DOM leakage was expressed as a fraction of the initial element or organic matter contents of aggregates and cultures. Maximum leakage measured at 60 to 100 MPa is given. Molar C:N ratios were calculated for aggregate and diatom biomass and for DOM leachates. To this end, the measured TDN concentrations were adjusted by −1 µM N to account for the minor concentrations of dissolved inorganic nitrogen (i.e., nitrate and ammonium) measured in the incubation vials, and thereby dissolved organic nitrogen (DON) concentrations were estimated. For aggregates, means ± SD of 21 aggregates incubated in five independent experiments are shown. For cultures, means ± SD of four independent pressure experiments are shown. ND, no data.

Diatom aggregates	Aggregate contents (μg/mm^3^)	Maximum leakage (μg/mm^3^)	Maximum leakage (%)
Total carbon	6.22 ± 1.32	2.97 ± 1.06	49.5 ± 17.5
Total nitrogen	0.87 ± 0.19	0.46 ± 0.23	57.7 ± 18.9
C:N ratio (M)	8.34 ± 2.54	7.69 ± 4.60	
***S. marinoi* cultures**	**Cellular contents (pg/cell)**	**Maximum leakage (pg/cell)**	**Maximum leakage (%)**
Total carbon	38.3 ± 0.4	16.1 ± 3.6	42.1 ± 9.5
Total nitrogen	4.2 ± 0.1	2.6 ± 0.5	63.0 ± 12.2
C:N ratio (M)	10.63 ± 0.37	7.37 ± 2.15	
Total lipids	19.5 ± 2.2	ND	ND
Total proteins	24.6 ± 9.7	11.3 ± 6.0	46.6 ± 15.1
Total carbohydrates	30.3 ± 2.8	25.9 ± 1.6	86.0 ± 11.9
Laminarin	29.4 ± 3.6	1.3 ± 0.4	4.5 ± 1.4
DMSP	2.18 ± 0.77	2.17 ± 0.09	99.6 ± 4.1

#### Molecular diversity of DOM

The biological formation and transformation index *I*_bio_ was calculated from molecular formulae of DOM present in the ambient seawater (Fig. 3F) and in the intracellular solutes of *S. marinoi* (Fig. 3L). The index considers specific molecular formulae in solid phase–extracted DOM and was deduced using mass spectral data covering a broad range from freshly microbially produced to aged and reworked oceanic DOM (*39*). High *I*_bio_ values are indicative of recent biological production of DOM that is presumably labile (*39*). According to this index, the intracellular DOM of *S. marinoi* can be considered labile and bioavailable to microbial heterotrophs (Fig. 3L). Pressure-induced DOM leakage from *S. marinoi* increased *I*_bio_ in the ambient seawater (Fig. 3F), which is consistent with the observed DOM concentration patterns.

#### Other diatom strains

Three additional diatom strains differing in cellular carbon and nitrogen contents by factors of ~20 and ~10, respectively, were used for experimentation (table S2). All three strains showed the same patterns of pressure-induced DOM leakage as observed for *S. marinoi* aggregates and cultures (fig. S1). However, the pressure level at which DOM leakage set in differed markedly between the three strains, i.e., at 20, 40, and 80 MPa for *Chaetoceros socialis*, *Conticribra weissflogii*, and *Phaeodactylum tricornutum*, respectively. Maximum DOC leakage corresponded to 17 to 42% of cellular carbon contents in all four diatom strains investigated (table S2). The loss of cellular nitrogen due to leakage was generally higher and amounted to 34 to 76%. Accordingly, the molar C:N ratio of the DOM leachates of the three strains was always lower than that of the biomass used for experimentation (table S2). Cell abundance did not vary in any of the four diatom strains throughout the incubations irrespective of the pressure treatment (fig. S2).

#### DOM leakage during pressurization versus depressurization

Incubations of *S. marinoi* cultures in an underpressure filtration module (fig. S3) confirmed that pressure-induced DOM leakage occurred during pressurization rather than depressurization (fig. S4). *S. marinoi* cultures filtered at pressure levels of 60 to 100 MPa (i.e., without a prior depressurization step) showed substantial increases in extracellular DOC and TDN concentrations, consistent with the depth-resolved patterns presented in Fig. 2. At the given cell density of 3.4 × 10^8^ cells/liter, the increase in extracellular DOC concentration corresponded to cell-specific leakage of 12.4 ± 3.1 pg of C per cell, which was at the lower end of the range of values presented in Table 1.

### DOM utilization experiment

#### Experimental simulation

**Fig. 4.**
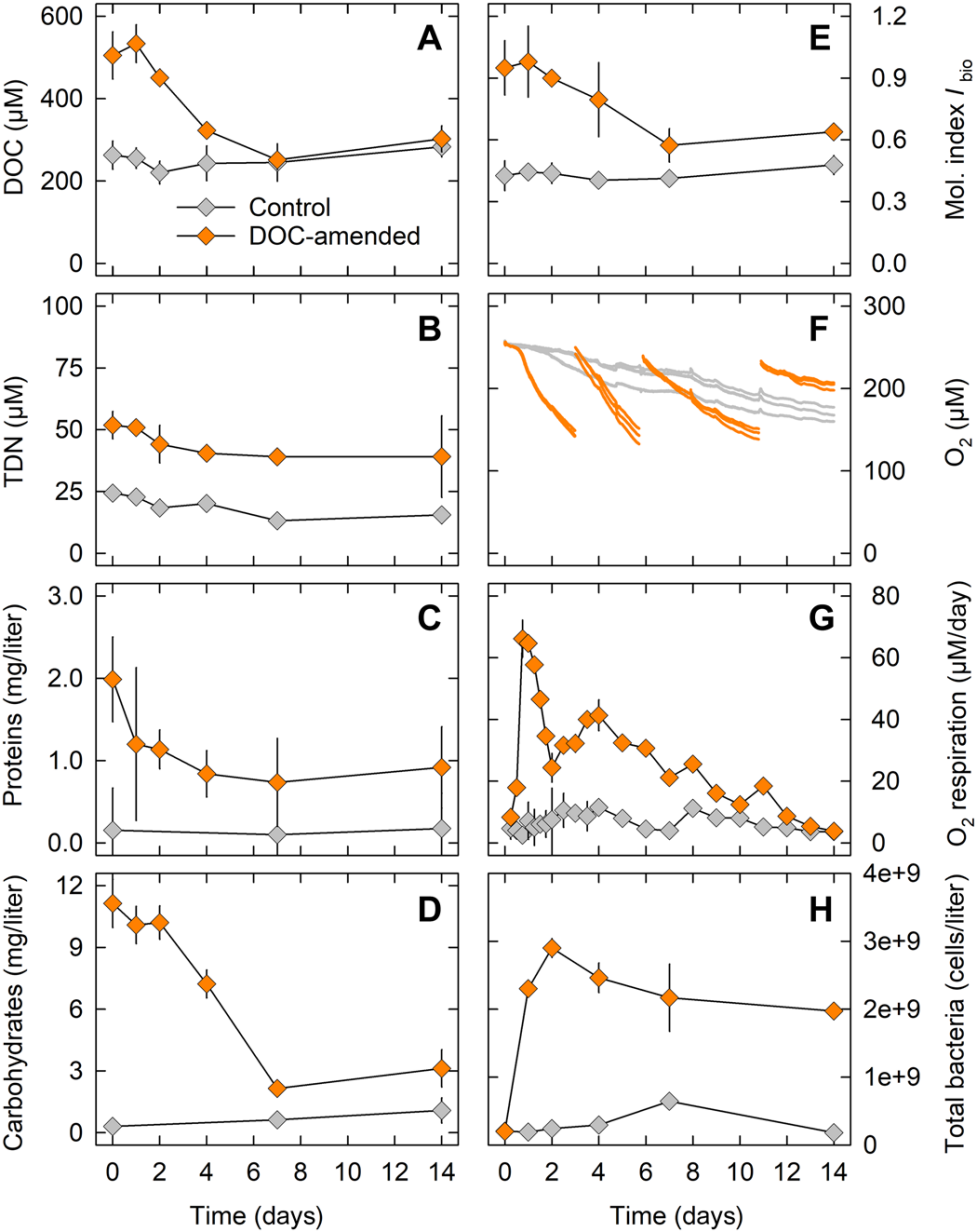
DOM utilization by a pelagic microbial community. Composition and utilization of leaked DOM added to a natural seawater microbial community incubated at atmospheric pressure and at 15°C for 2 weeks. (**A** to **H**) Time courses of DOC, TDN, total protein, total hydrolysable carbohydrate concentrations, the molecular index for biological formation and transformation (*I*_bio_), oxygen concentrations, aerobic respiration rates, and total bacterial abundance in DOM-amended versus control incubations are shown. Oxygen concentrations were measured in gas-tight vials that were re-aerated three times to maintain high oxygen levels. All other incubations were made in gas-permeable flasks in which oxygen levels always remained at >90% air saturation. Means ± SD of three replicate incubations are shown [in (F), individual replicates are shown].

Leaked DOM was produced by exposure of *S. marinoi* cultures to 60 MPa and 3°C for 24 hours (see Materials and Methods for details). The bioavailability of the leaked DOM for pelagic microbial communities was explored using a surface seawater microbial community incubated with or without leaked DOM (“DOM-amended” and control treatment, respectively) at 0.1 MPa and 15°C (Fig. 1). At the start of 2-week incubations, DOM amendment resulted in ~2-fold increases in DOC and TDN background concentrations and ~10-fold increases in protein and carbohydrate background concentrations (Fig. 4, A to D). The effect of DOM amendment on DOC, TDN, protein, and carbohydrate concentrations and on respiration rates and bacterial abundance was statistically significant (table S1).

#### DOM utilization and lability

All compound classes of leaked DOM were used by the microbial community mainly during the first week, while concentration changes were minor during the second week. Utilization of leaked TDN, proteins, and carbohydrates was incomplete; for bulk DOC, this could not be resolved within the error margins. The biological formation and transformation index *I*_bio_ had high values at the start of the experiment, decreased during the first week, and remained stable throughout the second week, although at slightly higher values than at the start of the incubation (Fig. 4E).

#### Microbial response

After a delay of ~12 hours, the microbial utilization of leaked DOM resulted in a strong decrease in oxygen concentration (Fig. 4F). Rates of aerobic community respiration peaked 18 to 24 hours after addition of leaked DOM and returned to ~70% lower rates within 24 hours (Fig. 4G). Respiration rates decreased over time in the DOM treatment but remained above those in the control treatment until day 12 of the incubation. Total bacterial abundance increased rapidly in the DOM treatment, peaked at ~30 times higher cell counts within 2 days, and remained relatively stable afterward (Fig. 4H). Notably, bacteria with a filamentous cell morphology increased in abundance by the end of the 2-week incubation (fig. S5).

## DISCUSSION

### Pressure-induced DOM leakage originates from diatoms

The parallel incubation of aggregates and cultures of the diatom *S. marinoi* allowed estimating the relative contribution of diatoms to total DOM leakage from sinking aggregates that are also colonized by bacteria and other microorganisms. By calculation, diatoms rather were the main and maybe exclusive contributors to DOC and TDN leakage. In contrast, assuming bacterial carbon contents typical of substrate-rich settings of 30 fg per cell (*40*) and the maximum bacterial abundance in model diatom aggregates (*33*), even completely solubilized bacteria would contribute as little as 0.075 μg of C/mm^3^ (i.e., ~3%) to DOC leakage from the aggregates.

In support of this quantitative argument, increasing extracellular concentrations of any given DOM constituent were always mirrored by decreasing intracellular concentrations of the same constituent. Different diatom strains leaked DOM at different pressure levels (e.g., *C. socialis* versus *P. tricornutum*), which was reflected in the loss of intracellular DOM at the respective pressure level. These observations indicate that pressure-exposed diatoms leak intracellular solutes from their cells into the surrounding seawater. The tentative intracellular localization of the leaking DOM is the cytoplasm and the vacuole of the diatoms because both DMSP (located in the cytoplasm) and laminarin (located in the vacuole) were leaking from the diatom cells under pressure. While the (sub)cellular mechanism of pressure-induced DOM leakage from diatoms is not yet known, it seems likely that it involves loss of cell membrane integrity, which, in future studies, might be tested using fluorescent probes (*41*). Impaired membranes may allow diffusion of cytoplasmic DMSP and vacuolar laminarin out of the cells, which normally might be hindered by their charge and size, respectively (*7*). The data obtained with the underpressure filtration module further show that pressure-induced DOM leakage is a relatively fast process on the order of minutes to hours. Mechanical squeezing of diatom cells by pressure must be minor, as water is compressed by only <4% at the given pressure and temperature levels (*42*). In accordance with this, light microscopy on pressure-exposed diatoms did not reveal broken frustules (*32*).

Pressure-induced DOM leakage appears widespread among diatoms. The four strains tested in this study covered both centric and pennate diatoms but consistently showed the same pressure response and only differed in the pressure level that triggered DOM leakage. It seems likely that other marine snow–forming microalgae are also prone to pressure-induced DOM leakage. Obvious candidates are dinoflagellates and fast-sinking coccolithophores, which both release DOM during bloom events (*43*, *44*) and potentially when exposed to high hydrostatic pressure.

### Composition of DOM leaking from diatoms

Dissolved proteins and carbohydrates were the dominant organic compound classes leaking from *S. marinoi* under pressure. Protein and carbohydrate leakage was accompanied by a strong increase in protein-like fluorescent DOM that is commonly considered to indicate the presence of relatively labile DOM (*45*). In contrast, humic-like fluorescent DOM, an indicator of relatively recalcitrant DOM (*23*, *24*), did not increase significantly. The dominance of proteins and carbohydrates reminds of the composition of photosynthetic exudates of eukaryotic microalgae in the surface ocean (*11*, *46*). Proteins were likely localized in the cytoplasm, whereas carbohydrates were likely localized in both the cytoplasm and the vacuole. Diatoms are known to accumulate (chryso)laminarin as their main storage polysaccharide. In marine snow sinking out of the photic zone, laminarin accounts for as much as 50% of the total particulate organic carbon (POC) contents (*47*), which makes it an important candidate for DOM leakage. Total hydrolysable carbohydrates and laminarin displayed different patterns of utilization and leakage under pressure: For total hydrolysable carbohydrates, the decrease in intracellular concentration equaled, within the error margins, the increase in extracellular concentration. However, for laminarin, the strong decrease in intracellular concentration was accompanied by only a weak increase in extracellular concentration. This implies internal laminarin utilization by surviving *S. marinoi* cells not only in the control but also in the pressure treatment, potentially to cover energy expenditures in darkness (*47*, *48*). As a consequence of the exhaustive internal laminarin utilization, substantial laminarin leakage could not occur at pressures of >20 MPa in the pressure-tank experiment. The laminarin contents of marine snow collected directly in the deep ocean remain unknown. Hence, future studies are needed to explore the contribution of laminarin and other intracellular molecules to carbon export and turnover in the deep ocean.

DMSP accounted for only a minor fraction of organic solutes in *S. marinoi*. The pattern of pressure-induced DMSP leakage was evident, with minimum intracellular and maximum extracellular concentrations precisely coinciding at 60 MPa. Our results imply that in *S. marinoi*, the potential role of DMSP as a piezolyte that protects the three-dimensional conformation of organic macromolecules at high pressure (*49*) can be exerted up to a maximum of 40 MPa. In *P. tricornutum*, intracellular DMSP was detectable up to a maximum of 60 MPa, while *C. weissflogii* and *C. socialis* did not contain any DMSP (see Data and materials availability; Stief, 2025a). Temperate diatoms typically produce little DMSP (*50*). This is in contrast to sea-ice diatoms, for which DMSP is recognized as a cryoprotectant (*51*), or dinoflagellate and haptophyte taxa that often contain one to two orders higher concentrations of intracellular DMSP. Further experiments are required to elucidate the depth-dependent leakage of DMSP in other taxa and to ascertain the suggested physiological function of DMSP in pressure-induced stress responses by eukaryotic phototrophs.

Pressure-induced DOM leakage might also include the release of lipids and nucleic acids from diatom cells or aggregates. While the total lipid contents of *S. marinoi* cultures were relatively high (this study), this compound class contributed <5% to the total carbon contents of diatom aggregates (*33*), suggesting a relatively low potential for lipid leakage under natural conditions. Similarly, nucleic acids contribute <5% to the total carbon contents of diatoms and other microalgae (*52*) and probably even less in the partially degraded diatom cells associated with sinking aggregates. Hence, the potential for nucleic acid leakage might also be relatively low. However, in contrast to proteins and carbohydrates, lipids and nucleic acids are particularly rich in phosphorus. It remains to be investigated whether pressure-induced DOM leakage represents a significant supply of phosphorus to the deep ocean.

### Functional role of DOM leaking from diatoms

Relatively high values of protein-like fluorescence and the molecular index *I*_bio_ and relatively low values of molar C:N ratios predicted that DOM leaking from pressure-exposed diatoms is labile. This prediction was corroborated in the DOM utilization experiment, as leaked DOM was readily available to heterotrophic pelagic microbes. All measured DOM constituents were rapidly used, although not completely, and DOM utilization was accompanied by increases in community respiration rate and bacterial abundance. Assuming bacterial carbon contents typical of substrate-rich settings of 30 fg of C per cell (*40*) and a respiratory quotient of 1, bacterial growth efficiency (BGE) in the DOM-amended treatment was estimated at 12% during the first 24 hours of incubation. This is in the range of BGEs determined for eutrophic coastal marine ecosystems and by far exceeds BGEs determined for oligotrophic ocean regions (*53*). Notably, *I*_bio_ decreased again in parallel with the concentrations of the used DOM constituents, supporting that microbial processing of labile DOM leaves recalcitrant DOM behind (*25*, *54*).

Our data do not resolve which specific organic compounds were used and which ones were not. However, carbohydrates were generally more efficiently used than proteins regarding rate and extent. This might be explained by the presence of laminarin in the leaked DOM used for the utilization experiment. In the marine pelagic, freely dissolved laminarin is rapidly used by heterotrophic microbes (*45*, *55*, *56*), and this was obviously also the case in the DOM utilization experiment. The high degradability of laminarin contrasts with that of structurally more complex polysaccharides, such as sulfated fucans, which are not abundant in *S. marinoi* (*57*, *58*).

In the marine pelagic, diatom-derived DOM fuels the microbial loop and thereby a minor fraction of DOM becomes available for higher trophic levels (*15*, *20*). In our DOM utilization experiment, bacterial abundance increased rapidly when the microbial community was amended with diatom-derived, leaked DOM. In addition, the dominance of different cell morphologies, such as filamentous types, changed dynamically during the 2-week incubation. It is thus obvious that the microbial community composition changed significantly in response to leaked DOM. However, our DOM utilization experiment was carried out with a surface microbial community incubated under surface-ocean pressure and temperature conditions. This approach enabled testing the bioavailability of leaked DOM as an inherent trait of DOM but did not reveal rate and extent of DOM degradation as influenced by origin of microbial community and pressure and temperature conditions. Future studies should thus investigate the response of deep-sea microbial communities to leaked DOM at the high-pressure and low-temperature levels prevailing at the depth of DOM leakage, ideally using in situ incubation systems (*59*, *60*).

### Conceptual considerations

**Fig. 5.**
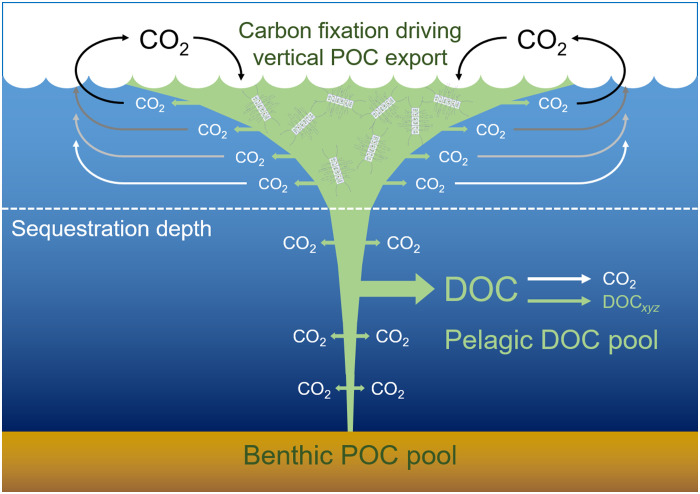
Conceptual scheme of pressure-induced leakage of DOC. At the ocean surface, primary producers (e.g., diatoms) fix carbon dioxide (CO_2_) into organic carbon that sustains the marine food web. Living and dead organisms represent particulate organic carbon (POC), some of which sinks down the water column as marine snow particles. The vertical POC export via the gravitational biological carbon pump is strongly attenuated because of microbial degradation and metazoan grazing, which produces CO_2_. If this process occurs close to the ocean surface, then CO_2_ is fixed again by primary producers or diffuses back into the atmosphere. POC degradation in the deep ocean proceeds at lower rates, and the CO_2_ produced is trapped below the sequestration depth (dashed line) for >100 years. Only a minor fraction of the sinking POC feeds the benthic POC pool and is sequestered in the seabed. At the high-pressure levels prevailing in the deep ocean, primary producers (e.g., diatoms) associated with fast-sinking “marine snow” particles leak DOC, some of which is metabolized by the pelagic microbiome of the deep ocean to form CO_2_ and/or diverse new DOC compounds (DOC*_xyz_*), the latter ones feeding the persistent pelagic DOC pool. Leaked DOC may thus be trapped as CO_2_ and/or DOC*_xyz_* below the sequestration depth for >100 years.

The results of this study imply a hitherto unrecognized pathway of the carbon cycle in the deep-ocean, pressure-induced DOM leakage (Fig. 5). Diatoms and potentially other microalgae associated with rapidly sinking particles leak DOM when they are exposed to high-pressure levels. On the one hand, this process represents a carbon loss for the biological carbon pump and thus contributes to the attenuation of the vertical POC flux in the deep ocean (*61*, *62*). Pressure-induced DOM leakage thus supplements the other known pathways of flux attenuation, i.e., microbial degradation of and metazoan grazing on sinking particles (*3*). The decreasing amounts of POC collected by sediment traps at greater water depths likely result from the combined effects of any of these three pathways. On the other hand, pressure-induced DOM leakage supplies labile, bioavailable carbon to the pelagic microbiome of the deep ocean. Supply of labile DOM to the deep ocean has recently been ascribed to migrating zooplankton, but this is limited to the upper ~2 km (*63*). In contrast, pressure-induced DOM leakage occurs at depths of 2 to 6 km and thus well below the sequestration depth for organic and inorganic carbon (*27*–*29*). Hence, this process will be of particular importance when fast-sinking particles dominate the vertical POC flux or when particle export is mediated by the recently described “particle injection pumps” that bypass the gravitational biological carbon pump (*64*). Either way, DOM leaking from sinking particles might be stored in the deep ocean for centuries, irrespective of whether it is microbially mineralized to CO_2_ or transformed into other dissolved organic compounds. Microbial processing and diversification of DOM derived from pressure-induced leakage will likely increase its recalcitrance and sequestration time in the deep ocean (*22*, *26*, *53*). Pressure-induced DOM leakage may thus fuel the persistent DOM pool in the bathypelagic and abyssopelagic zones.

Beyond the biogeochemical implications of pressure-induced DOM leakage, this pathway of particle solubilization has as yet unknown biological and ecological dimensions. We envisage that leaked DOM will fuel the microbial loop and thus the planktonic food web in the deep ocean (*15*, *20*). This process likely entails shifts in microbial community composition that might or might not resemble the succession patterns of bacterioplankton induced by diatom blooms in the surface ocean (*55*). Exploring these succession patterns in the deep ocean means to entering uncharted territory, as the key players in the degradation of labile, diatom-derived DOM still remain to be found in the largest habitat of the biosphere.

## MATERIALS AND METHODS

### Diatom cultures and model diatom aggregates

#### Diatoms

Four diatom strains were obtained from the culture collections National Center for Marine Algae and Microbiota (NCMA; Bigelow, USA), Roscoff Culture Collection (Station Biologique de Roscoff, France), and Gothenburg University Microalgae Culture Collection (GUMACC; University of Gothenburg, Sweden): *S. marinoi* (CCMP 1332), *C. weissflogii* (formerly known as *Thalassiosira weissflogii*) (CCMP 1336), *C. socialis* (RCC 6904), and *P. tricornutum* (GUMACC 2) (see Data and materials availability; Stief, 2025a). The strains were cultured in L1 medium plus silicate (*65*) prepared with sterilized natural seawater [35 practical salinity unit (psu)]. The cultures were grown at 15°C under a light/dark regime of 14/10 hours. Before each experiment, diatom exudates in the culture flasks were removed by a washing step: The cultures were centrifuged at 500*g* for 5 min, the supernatant was discarded, and the cells were resuspended in sterile seawater media (35 psu) prepared from Red Sea Salt (Red Sea Aquatics Ltd., Cheddar, UK) and deionized tap water (“RSW35”). Background DOC and TDN concentrations of RSW35 were ~150 μM C and ~30 μM N, respectively. Cellular carbon and nitrogen contents of each diatom strain were determined in five replicates of known cell abundance using a Na 1500 elemental analyzer (Fisons, Loughborough, UK).

#### Aggregates

Model diatom aggregates were produced in 600-ml glass bottles rotating on a plankton wheel (*32*, *66*) (see Data and materials availability; Stief, 2025b). To this end, 100 ml of stationary-phase *S. marinoi* culture was diluted with 500 ml of 3-μm filtered surface seawater collected in Øresund off Denmark. Near-spherical aggregates formed within 1 to 3 days. Before using the aggregates in pressure-tank experiments, they were individually transferred to RSW35 to wash off diatom exudates and microorganisms not attached to the aggregates. Diatom cell counts and sizing of diatom aggregates were performed, as described in (*33*).

### DOM leakage experiments

#### Simulation of marine snow sinking into the deep ocean

To explore pressure-induced DOM leakage from marine snow, 10 rotating incubation tanks were used to simulate the sinking of diatom cells and aggregates from the surface ocean into a deep-sea trench within 20 days (Fig. 1) (*32*, *33*). The horizontal rotation of the cylindrical tanks kept diatom cells and aggregates in suspension as if they were sinking through the water column. In five incubation tanks, hydrostatic pressure was incrementally increased by 5 MPa/day to simulate the sinking speed of 500 m/day previously determined for model diatom aggregates (*32*) (pressure treatment). The pressure increase was administered every 24 hours at a speed of 1 MPa/min. After 20 days, the maximum pressure of 100 MPa was reached, which corresponds to a water depth of 10 km. In five incubation tanks, hydrostatic pressure was not increased during the 20-day incubation (control treatment). Every 4 days, one pressure and one control tank were opened to retrieve samples for analysis. This incubation and sampling scheme allowed to reconstruct depth profiles of pressure effects on different variables at 2-km resolution (i.e., at 0, 2, 4, 6, 8, and 10 km of simulated sinking depth). Since temperature critically determines membrane fluidity and functionality and thus potentially also DOM leakage from diatoms, the incubation temperature during the DOM leakage experiments was 3°C, which is in the range of deep-ocean temperatures.

Inside the pressure and control tanks, diatom cultures were incubated in acid-washed 32-ml polyethylene flasks, while diatom aggregates were individually incubated in acid-washed 5.9-ml glass exetainers (Labco Ltd., Lampeter, UK). The flexible polyethylene wall of the flasks and the chlorobutyl rubber septum of the exetainers efficiently transmit the hydrostatic pressure level inside the tanks into the actual incubation vials. The flexible septum prohibits the glass exetainers from breaking under pressure.

#### Sampling scheme

For each of the four diatom cultures and for the diatom aggregates, the pressure experiment was repeated four and five times, respectively. Upon sample retrieval (including at time 0), the diatom cultures were first sampled for cell abundance by fixing 1 ml of cell suspension with 50 μl of 37% formaldehyde. The remainder of the culture was filtered over a precombusted 0.3-μm glass fiber filter (GF75, Advantec MFS Inc., Dublin, USA). The filtrate was split into 3 ml for combined DOC and TDN analysis, 10 ml for fluorescence measurements, 1 ml each for total protein, total carbohydrate, and DMSP analysis, 5 ml for enzyme-based laminarin analysis, and 10 ml for ultrahigh-resolution mass spectrometric DOM analysis. The split filtrates were stored at −20°C, except for the DOC, TDN, and DOM fluorescence samples that were kept at 4°C and analyzed within 1 day (DOC and TDN) and 1 week (DOM fluorescence).

The diatom biomass retained on the filter was freeze dried and split into subsamples for analyzing the intracellular contents of DOC and TDN (combined sample), total protein, total carbohydrate, laminarin, and DMSP, as well as the molecular composition of DOM. The variables fluorescence, laminarin, DMSP, and DOM composition were each analyzed in one experiment only. For the analysis of intracellular DOC, TDN, and DOM composition, the diatom cells retained on filters were lysed through rapid freeze-thaw cycling, as described in (*67*). In contrast, the analyses of intracellular proteins, carbohydrates, laminarin, and DMSP involved extraction or lysis steps in the respective analytical procedure (see below).

The diatom aggregates were individually sized through photographing and image analysis before the experimental incubations (*33*). In addition, for each experiment, a subset of aggregates was analyzed for diatom cell abundance and total carbon and nitrogen contents. Upon sample retrieval during the experimental incubation (including at time 0), the diatom aggregates were allowed to settle onto the bottom of the exetainers. The top 4 ml of seawater in the exetainers was carefully siphoned off and filtered through a precombusted 0.3-μm glass-fiber filter (GF75) and used for combined DOC and TDN analysis. For fluorescence measurements, filtered seawater from three exetainers was pooled to arrive at a sample volume of 10 ml.

#### DOM leakage during pressurization versus depressurization

To clarify whether pressure-induced DOM leakage from diatoms occurs during pressurization or depressurization, *S. marinoi* cultures were incubated in a custom-made, underpressure filtration module (fig. S3). In this module, two 20-ml glass syringes were tightly connected via a filter cartridge loaded with a precombusted 0.3-μm glass-fiber filter (GF75). One of the two syringes was filled with washed *S. marinoi* culture, while the other syringe remained empty. The connected syringes were mounted onto the module with the piston of the diatom-filled syringe fastened to a spring-operated fixture. The filtration module holding in total three pairs of syringes was inserted into a pressure tank equipped with an electrical feedthrough in the lid (*32*). In serial incubations, the tank was pressurized to 0.1, 20, 40, 50, 60, 80, or 100 MPa at a speed of 1 MPa/min and horizontally rotated to keep the diatom cells in suspension (*32*). The pressure level adjusted inside the tank was transmitted into the diatom-filled syringe via the movable piston. After 30 min of pressure exposure, the three pairs of syringes were individually released by electric signals sent through the feedthrough in the lid of the pressure tank. Thereby, the contents of the diatom-filled syringes were pressed through the glass-fiber filters, which physically separated the diatom biomass retained on the filter from the seawater in the receiving syringe. Only after filtration was completed in all three pairs of syringes (~30 s each), the tank was depressurized (~10 min), and the syringes were retrieved for sampling (~5 min). Any DOM that had leaked from the diatoms during pressurization was contained in the filtered seawater, whereas any DOM leaked during the subsequent depressurization did not get in contact with the filtered seawater.

### DOM utilization experiment

#### Experimental design

Bioavailability and degradation of DOM leaking from pressure-exposed *S. marinoi* were studied in surface seawater collected in the German Bight before the onset of the spring bloom and stored in darkness until used for experimentation (see Data and materials availability; Stief 2025c) (Fig. 1). The seawater was 3-μm filtered before use. DOM was produced by exposing ~180 ml of washed culture of *S. marinoi* to 60 MPa and 3°C for 24 hours to induce DOM leakage. Following depressurization, the *S. marinoi* culture was filtered through a precombusted 0.3-μm glass-fiber filter (GF75) and the filtrate used for experimentation. Three acid-washed 140-ml polyethylene flasks were filled with one part of DOM and two parts of 3-μm filtered surface seawater (DOM-amended treatment), leaving a 10-ml headspace in the flask. Three acid-washed 140-ml polyethylene flasks were filled with one part of 0.3-μm filtered and two parts of 3-μm filtered surface seawater (control treatment) to arrive at the same dilution of the microbial community in both treatments. To provide conditions conducive of active DOM degradation by the surface microbial community, the seawater-filled flasks were incubated at 0.1 MPa and 15°C.

#### Sampling scheme

The six flasks were incubated on a horizontal shaker for 2 weeks during which they were sampled six times. At each time point, the flasks were first sampled for bacterial cell abundance by fixing 1 ml of seawater with 50 μl of 37% formaldehyde. Then, 15 ml of seawater was collected and 0.3-μm filtered (GF75), and the filtrate was split into 3 ml for combined DOC and TDN analysis, 1 ml each for total protein and total carbohydrate analysis, and 10 ml for ultrahigh-resolution mass spectrometric DOM analysis. The split filtrates were stored at −20°C until analysis.

#### Respiration activity

To continuously measure the respiration activity of the microbial communities, a proxy for oxidative carbon mineralization, two sets of three 5.9-ml exetainers equipped with an oxygen-sensing optode foil were filled with DOM-amended and nonamended (control) surface seawater (*32*). The exetainers were placed on a horizontal shaker and connected to a computer-controlled oxygen meter (FireSting, PyroScience, Aachen, Germany). The observed changes in oxygen concentration over time were used to calculate respiration rates for defined time intervals. To avoid oxygen depletion in the seawater, the exetainers were re-aerated three times, as described in (*68*). At each sampling time point in the 140-ml polyethylene flasks, oxygen measurements revealed air saturation levels of >90%, presumably because of the headspace in the flasks and the gas permeability of polyethylene.

### DOM analyses

#### DOC and TDN

Extra- and intracellular DOC and TDN samples were analyzed on a total organic carbon (TOC) analyzer (TOC-L, Shimadzu Europa GmbH). TDN is the sum of dissolved organic nitrogen (DON) and dissolved inorganic nitrogen, i.e., ammonium, nitrite, and nitrate.

#### DOM fluorescence

Absorbance and fluorescence of DOM were determined using a HORIBA Aqualog at an excitation between 240 and 600 nm (increment 5 nm) and an emission between 246 and 824 nm (increment ~4.6 nm). The wavelength accuracy of the excitation monochromator and the emission detector (±1 nm), along with cuvette immaculacy were verified daily before analytical measurements (*69*). An ultrapure water sample (Starna Scientific Ltd.) was used as the blank and to determine the instrument-specific Raman scatter at an excitation of 350 nm. Arbitrary fluorescence counts were divided by this area; fluorescence intensities were reported in Raman units. After the subtraction of the blank, Raman and Rayleigh scatters were removed without interpolation and inner-filter effects compensated with the absorbance-based approach (*70*). Protein-like fluorescence (peak “T”) was extracted at excitation and emission wavelengths of 275 and 340 nm, respectively, whereas humic-like fluorescence (peak “D”) was extracted at 390 and 509 nm, respectively (*71*, *72*). The reported peak T was highly correlated with peaks “M” and “C” (*R*^2^ ~ 0.99) (*71*), while the reported peak D was significantly correlated with peak “A” (*R*^2^ = 0.95) (*71*).

#### Total proteins

Proteins were extracted and hydrolyzed in 0.5 M NaOH at 80°C for 30 min (*73*). This was achieved by adding 0.5 ml of 1.5 M NaOH to 1-ml seawater samples and 3 ml of 0.5 M NaOH to filter sections of known size. After cooling, the samples were centrifuged at 3000*g* for 10 min and analyzed by the Lowry protein assay (*74*). Bovine serum albumin was used for preparing calibration standards (0 to 50 mg/liter).

#### Total carbohydrates

Hydrolysable carbohydrates were quantified by total acid hydrolysis in 1 M HCl at 100°C for 24 hours (*75*). This was achieved by combining 1 ml of 2 M HCl with 1-ml seawater samples or by adding 3 ml of 1 M HCl to filter sections of known size in sealed glass vials. After cooling, the samples were centrifuged at 3000*g* for 10 min and analyzed by the 4-hydroxybenzoic acid hydrazide (PAHBAH) reducing sugar assay (*76*, *77*). Since laminarin was identified as the major polysaccharide in diatom-derived particles (*47*), we used laminarin extracted from *Laminaria digitata* (L9634, Sigma-Aldrich, Germany) for preparing calibration standards (0 to 200 mg/liter).

#### Laminarin

Intracellular laminarin was extracted from filter sections of known size with 50 mM Mops buffer at 60°C for 1 hour (*75*). Extracellular laminarin was desalted by dialysis of 10 ml of filtrate in 1-kDa tubing (Spectra/Por, Spectrum Laboratories) against 10 liters of Milli-Q water with stirring and then concentrated by freeze-drying and resuspension in 0.45 ml of Milli-Q water. Laminarin standards from *L. digitata* (L9634, Sigma-Aldrich, Germany) were dialyzed and freeze dried in parallel with the samples. Extra- and intracellular laminarin were quantified in technical triplicates by specific hydrolysis with laminarinases and the PAHBAH reducing sugar assay following (*75*, *77*).

#### Dimethylsulfoniopropionate

DMSP dissolved in seawater (extracellular DMSP) and intracellular DMSP (IC-DMSP) were quantified using headspace direct injection of gaseous phase (for IC-DMSP) and the in-vial purging of aqueous phase (for extracellular DMSP) methodologies described in (*78*). Briefly, 1 ml of seawater or one filter section of known size were transferred into a 3-ml glass exetainer sealed with a polytetrafluoroethylene-coated rubber septum (Labco Ltd., Lampeter, UK). DMSP was hydrolyzed to dimethyl sulfide (DMS) by injecting 1 ml of 1 N NaOH through the vial septum, followed by incubation at room temperature for at least 1 day. Samples were equilibrated at 26°C overnight before gas chromatographic quantification with flame photometric detection of DMS (Shimadzu GC-2010, Milton Keynes, UK) using a 60 m–by–0.53 mm–by–7 μm capillary column (RTX-1, Thames Restek UK, Saunderton, UK). The gas chromatograph was calibrated (*R*^2^ > 0.99) with DMSP synthesized from DMS and acrylic acid as described in (*79*). The limits of quantification were 600 pmol of DMS (lowest calibration concentration for direct injection method) and 3.6 ± 1.86 pmol of DMS (10 times peak height above baseline for in-vial purge method).

#### Ultrahigh-resolution mass spectrometry of DOM

After thawing, samples were immediately acidified to pH 2 (hydrochloric acid, 25%, analytical grade) and extracted via 100 mg of Priority PolLutant (PPL) resin (Agilent Bond Elut) following the procedure of (*80*). PPL reproducibly extracts a well-defined fraction of DOM, covering a broad range of polarities and molecule sizes. Small polar compounds are not quantitatively retained on PPL resin, with extraction efficiencies for seawater of ~60%. Solid phase–extracted DOM was eluted with ~0.5 ml of methanol (high-performance liquid chromatography grade), and procedural blanks were prepared with ultrapure water for each set of processed samples. For analysis on a 15 Tesla SolariX Fourier transform ion cyclotron resonance mass spectrometry (FT-ICR-MS; Bruker Daltonik GmbH, Bremen, Germany), extracts were diluted to reach similar organic carbon concentrations in 1:1 methanol:water, ensuring maximum comparability of mass spectra. Ionization was achieved via electrospray in negative mode. Ion accumulation time was 0.6 s, and 200 scans were accumulated per sample. All samples were analyzed in technical duplicates, and deep-sea DOM reference material (https://uol.de/en/icbm/dsr-dom) was repeatedly analyzed together with the samples for quality and instrument performance control. Mass spectra were calibrated with a list of exact masses of confirmed molecular formulae to achieve <0.1 parts per million of mass error over the considered mass/charge ratio range of 100 to 800. Calibrated spectra were then processed with the server-based tool ICBM-OCEAN (*81*) to align detected masses and assign molecular formulae. For further data analysis, a minimum detection limit of 3 and the “likeliest match” output file were chosen. The index for biological formation and transformation (*I*_bio_) was calculated from the peak intensities of specific molecular formulae and provides a measure for recent biological processing of DOM (*39*). More specifically, *I*_bio_ is the ratio of the sum of peak intensity of five known molecular formulae that indicate recent biological processing of DOM and of five known molecular formulae that are not influenced by recent biological processing of DOM. High *I*_bio_ values suggest recent bioproduction of DOM of presumably high lability.

### Microbial abundance

For each sampling time point of the 2-week DOM utilization experiment, the abundance of prokaryotes (bacteria and archaea) was determined through automated microscopic cell counts and image analysis using ACMEtool3 (*82*). The 1-ml seawater samples were filtered onto 0.2-μm polycarbonate membranes (Isopore, Merck, Germany), washed with 10 ml of 0.2-μm filtered Milli-Q water, and stored at −20°C. One section of each membrane filter was embedded in Citifluor:VECTASHIELD (3:1) that contained 4′,6-diamidino-2-phenylindole (DAPI; 1 μg/ml; Sigma-Aldrich, Germany) for nucleic acid staining. Images were automatically acquired on a Zeiss AxioImager. Z2 microscopic stand (Carl Zeiss MicroImaging GmbH, Germany) with a cooled charge-coupled device camera (AxioCam MRm, Carl Zeiss) and a Colibri light-emitting diode light source (Carl Zeiss) with a light-emitting diode (365 ± 4.5 nm) for DAPI (*82*).

### Statistical analysis

After testing for normal distribution and homogeneity of variance of the replicated data, repeated-measures analysis of variance (ANOVA) was used to test the vertical profiles and the time series of the different variables analyzed in the DOM leakage and utilization experiments, respectively, for statistically significant differences. For the DOM leakage experiments, the main effects tested were “pressure treatment” and “time” and the interaction between the two. For the DOM utilization experiments, the main effects tested were “DOM amendment” and time and the interaction between the two. Nonreplicated data were not statistically tested. Statistical testing was made in SigmaPlot 13 (Grafiti LLC, Palo Alto, USA). The results are summarized in table S1.

## References

[1] L. Alldredge, M. W. Silver, Characteristics, dynamics and significance of marine snow. Prog. Oceanogr. 20, 41–82 (1988).

[2] M. Simon, H. P. Grossart, B. Schweitzer, H. Ploug, Microbial ecology of organic aggregates in aquatic ecosystems. Aquat. Microb. Ecol. 28, 175–211 (2002).

[3] M. H. Iversen, Carbon export in the ocean: A biologist’s perspective. Ann. Rev. Mar. Sci. 15, 357–381 (2023). 10.1146/annurev-marine-032122-035153 36055975

[4] D. J. Burdige, Preservation of organic matter in marine sediments: Controls, mechanisms, and an imbalance in sediment organic carbon budgets? Chem. Rev. 107, 467–485 (2007). 17249736 10.1021/cr050347q

[5] G. A. Jackson, D. M. Checkley Jr., Particle size distributions in the upper 100 m water column and their implications for animal feeding in the plankton. Deep Sea Res. 1 Oceanogr. Res. Pap. 58, 283–297 (2011).

[6] U. Alcolombri, F. J. Peaudecerf, V. I. Fernandez, L. Behrendt, K. S. Lee, R. Stocker, Sinking enhances the degradation of organic particles by marine bacteria. Nat. Geosci. 14, 775–780 (2021).

[7] M. S. Weiss, U. Abele, J. Weckesser, W. Welte, E. Schiltz, G. E. Schulz, Molecular architecture and electrostatic properties of a bacterial porin. Science 254, 1627–1630 (1991). 1721242 10.1126/science.1721242

[8] N. Jiao, G. J. Herndl, D. A. Hansell, R. Benner, G. Kattner, S. W. Wilhelm, D. L. Kirchman, M. G. Weinbauer, T. Luo, F. Chen, F. Azam, Microbial production of recalcitrant dissolved organic matter: Long-term carbon storage in the global ocean. Nat. Rev. Microbiol. 8, 593–599 (2010). 20601964 10.1038/nrmicro2386

[9] C. A. Carlson, D. A. Hansell, “DOM sources, sinks, reactivity, and budgets,” in *Biogeochemistry of Marine Dissolved Organic Matter*, D. A. Hansell, C. A. Carlson, Eds. (Elsevier Science & Technology, ProQuest Ebook, 2015), pp. 65–126.

[10] B. Biddanda, R. Benner, Carbon, nitrogen, and carbohydrate fluxes during the production of particulate and dissolved organic matter by marine phytoplankton. Limnol. Oceanogr. 42, 506–518 (1997).

[11] C. Romera-Castillo, H. Sarmento, X. A. Alvarez-Salgado, J. M. Gasol, C. Marrasé, Production of chromophoric dissolved organic matter by marine phytoplankton. Limnol. Oceanogr. 55, 446–454 (2010).

[12] C. A. Suttle, Viruses in the sea. Nature 437, 356–361 (2005). 16163346 10.1038/nature04160

[13] C. F. Kranzler, D. A. Busono, G. J. Walsh, A. C. Carrillo, K. D. Bidle, K. Thamatrakoln, Taxonomically distinct diatom viruses differentially impact microbial processing of organic matter. Sci. Adv. 11, eadq5439 (2025). 40315326 10.1126/sciadv.adq5439 PMC12047433

[14] F. Møller, P. Thor, T. G. Nielsen, Production of DOC by *Calanus finmarchicus*, *C. glacialis* and *C. hyperboreus* through sloppy feeding and leakage from fecal pellets. Mar. Ecol. Prog. Ser. 262, 185–191 (2003).

[15] B. C. Cho, F. Azam, Major role of bacteria in biogeochemical fluxes in the ocean’s interior. Nature 332, 441–443 (1988).

[16] D. C. Smith, M. Simon, A. L. Alldredge, F. Azam, Intense hydrolytic enzyme activity on marine aggregates and implications for rapid particle dissolution. Nature 359, 139–142 (1992).

[17] T. Kiørboe, G. A. Jackson, Marine snow, organic solute plumes, and optimal chemosensory behavior of bacteria. Limnol. Oceanogr. 46, 1309–1318 (2001).

[18] C. Tamburini, J. Garcin, A. Bianchi, Role of deep-sea bacteria in organic matter mineralization and adaptation to hydrostatic pressure conditions in the NW Mediterranean Sea. Aquat. Microb. Ecol. 32, 209–218 (2003).

[19] J. T. Turner, Zooplankton fecal pellets, marine snow, phytodetritus and the ocean’s biological pump. Prog. Oceanogr. 130, 205–248 (2015).

[20] F. Azam, T. Fenchel, J. G. Field, J. S. Gray, L.-A. Meyer-Reil, F. Thingstad, The ecological role of water-column microbes in the sea. Mar. Ecol. Prog. Ser. 10, 257–263 (1983).

[21] B. E. Noriega-Ortega, G. Wienhausen, A. Mentges, T. Dittmar, M. Simon, J. Niggemann, Does the chemodiversity of bacterial exometabolomes sustain the chemodiversity of marine dissolved organic matter? Front. Microbiol. 10, 215 (2019). 30837961 10.3389/fmicb.2019.00215 PMC6382689

[22] T. Dittmar, S. T. Lennartz, “Reasons behind the long-term stability of dissolved organic matter,” in *Biogeochemistry of Marine Dissolved Organic Matter* (Academic Press, 3rd ed., 2024), pp. 613–655.

[23] Y. Yamashita, E. Tanoue, Production of bio-refractory fluorescent dissolved organic matter in the ocean interior. Nat. Geosci. 1, 579–582 (2008).

[24] L. Jørgensen, C. A. Stedmon, M. A. Granskog, M. Middelboe, Tracing the long-term microbial production of recalcitrant fluorescent dissolved organic matter in seawater. Geophys. Res. Lett. 41, 2481–2488 (2014).

[25] L. Legendre, R. B. Rivkin, M. G. Weinbauer, L. Guidi, J. Uitz, The microbial carbon pump concept: Potential biogeochemical significance in the globally changing ocean. Prog. Oceanogr. 134, 432–450 (2015).

[26] T. Dittmar, S. T. Lennartz, H. Buck-Wiese, D. A. Hansell, C. Santinelli, C. Vanni, B. Blasius, J.-H. Hehemann, Enigmatic persistence of dissolved organic matter in the ocean. Nat. Rev. Earth Environ. 2, 570–583 (2021).

[27] R. S. Lampitt, E. P. Achterberg, T. R. Anderson, J. A. Hughes, M. D. Iglesias-Rodriguez, B. A. Kelly-Gerreyn, M. Lucas, E. E. Popova, R. Sanders, J. G. Shepherd, D. Smythe-Wright, A. Yool, Ocean fertilization: A potential means of geoengineering? Philos. Trans. R. Soc. Lond. A 366, 3919–3945 (2008). 10.1098/rsta.2008.0139 18757282

[28] U. Passow, C. A. Carlson, The biological carbon pump in a high CO_2_ world. Mar. Ecol. Prog. Ser. 470, 249–271 (2012).

[29] C. A. Baker, A. P. Martin, A. Yool, E. Popova, Biological carbon pump sequestration efficiency in the North Atlantic: A leaky or a long-term sink? Global Biogeochem. Cycles 36, e2021GB007286 (2022).

[30] C. M. Turley, The effect of pressure on leucine and thymidine incorporation by free-living bacteria and by bacteria attached to sinking oceanic particles. Deep Sea Res. I Oceanogr. Res. Pap. 40, 2193–2206 (1993).

[31] C. Tamburini, M. Boutrif, M. Garel, R. R. Colwell, J. W. Deming, Prokaryotic responses to hydrostatic pressure in the ocean – A review. Environ. Microbiol. 15, 1262–1274 (2013). 23419081 10.1111/1462-2920.12084

[32] P. Stief, M. Elvert, R. N. Glud, Respiration by “marine snow” at high hydrostatic pressure: Insights from continuous oxygen measurements in a rotating pressure tank. Limnol. Oceanogr. 66, 2797–2809 (2021). 34413544 10.1002/lno.11791 PMC8359982

[33] P. Stief, C. Schauberger, K. W. Becker, M. Elvert, J. P. Balmonte, B. Franco-Cisterna, M. Middelboe, R. N. Glud, Hydrostatic pressure induces transformations in the organic matter and microbial community composition of marine snow particles. Commun. Earth Environ. 4, 377 (2023).

[34] B. Franco-Cisterna, P. Stief, R. N. Glud, Hydrostatic pressure impedes the degradation of sinking copepod carcasses and fecal pellets. J. Plankton Res. 46, 219–223 (2024). 38572121 10.1093/plankt/fbae002 PMC10987097

[35] C. Tamburini, J. Garcin, G. Gregori, K. Leblanc, P. Rimmelin, D. L. Kirchman, Pressure effects on surface Mediterranean prokaryotes and biogenic silica dissolution during a diatom sinking experiment. Aquat. Microb. Ecol. 43, 267–275 (2006).

[36] C. C. Lloyd, J. P. Balmonte, R. N. Glud, C. Arnosti, Strong effects of increased hydrostatic pressure on polysaccharide-hydrolyzing enzyme activities in coastal seawater and sediments. J. Geophys. Res. Biogeosci. 130, e2024JG008417 (2025).

[37] R. N. Glud, J. K. Gundersen, B. B. Jørgensen, N. P. Revsbech, H. D. Schulz, Diffusive and total oxygen uptake of deep-sea sediments in the eastern South Atlantic Ocean: In situ and laboratory measurements. Deep Sea Res. I Oceanogr. Res. Pap. 41, 1767–1788 (1994).

[38] P. O. J. Hall, J. Brunnegård, G. Hulthe, W. R. Martin, H. Stahl, A. Tengberg, Dissolved organic matter in abyssal sediments: Core recovery artifacts. Limnol. Oceanogr. 52, 19–31 (2007).

[39] S. K. Bercovici, M. Wiemers, T. Dittmar, J. Niggemann, Disentangling biological transformations and photodegradation processes from marine dissolved organic matter composition in the global ocean. Environ. Sci. Technol. 57, 21145–21155 (2023). 38065573 10.1021/acs.est.3c05929 PMC10734261

[40] R. Fukuda, H. Ogawa, T. Nagata, I. Koike, Direct determination of carbon and nitrogen contents of natural bacterial assemblages in marine environments. Appl. Environ. Microbiol. 64, 3352–3358 (1998). 9726882 10.1128/aem.64.9.3352-3358.1998 PMC106732

[41] D. C. O. Thornton, J. Chen, Exopolymer production as a function of cell permeability and death in a diatom (*Thalassiosira weissflogii*) and a cyanobacterium (*Synechococcus elongatus*). J. Phycol. 53, 245–260 (2017). 27690180 10.1111/jpy.12470

[42] R. A. Fine, F. J. Millero, Compressibility of water as a function of temperature and pressure. J. Chem. Phys. 59, 5529–5536 (1973).

[43] A. Engel, B. Delille, S. Jacquet, U. Riebesell, E. Rochelle-Newall, A. Terbrüggen, I. Zondervan, Transparent exopolymer particles and dissolved organic carbon production by *Emiliania huxleyi* exposed to different CO_2_ concentrations: A mesocosm experiment. Aquat. Microb. Ecol. 34, 93–104 (2004).

[44] S. Elovaara, E. Eronen-Rasimus, E. Asmala, T. Tamelander, H. Kaartokallio, Contrasting patterns of carbon cycling and dissolved organic matter processing in two phytoplankton-bacteria communities. Biogeosciences 18, 6589–6616 (2021).

[45] Y. Yamashita, E. Tanoue, Chemical characterization of protein-like fluorophores in DOM in relation to aromatic amino acids. Mar. Chem. 82, 255–271 (2003).

[46] S. M. Myklestad, “Dissolved organic carbon from phytoplankton,” in *The Handbook of Environmental Chemistry Vol. 5, Part D, Marine Chemistry*, P. Wangersky, Ed. (Springer, 2000), pp. 111–148.

[47] S. Becker, J. Tebben, S. Coffinet, K. Wiltshire, M. H. Iversen, T. Harder, K.-U. Hinrichs, J.-H. Hehemann, Laminarin is a major molecule in the marine carbon cycle. Proc. Natl. Acad. Sci. U.S.A. 117, 6599–6607 (2020). 32170018 10.1073/pnas.1917001117 PMC7104365

[48] A. Kamp, D. de Beer, J. L. Nitsch, G. Lavik, P. Stief, Diatoms respire nitrate to survive dark and anoxic conditions. Proc. Natl. Acad. Sci. U.S.A. 108, 5649–5654 (2011). 21402908 10.1073/pnas.1015744108 PMC3078364

[49] P. H. Yancey, Compatible and counteracting solutes: Protecting cells from the Dead Sea to the deep sea. Sci. Prog. 87, 1–24 (2004). 15651637 10.3184/003685004783238599 PMC10367508

[50] M. D. Keller, W. K. Bellows, R. R. L. Guillard, “Dimethylsulfide production in marine phytoplankton,” in *Biogenic Sulfur in the Environment*, E. S. Saltzman, W. J. Cooper, Eds. (American Chemical Society, 1989), pp. 167–182.

[51] M. Levasseur, Impact of Arctic meltdown on the microbial cycling of sulphur. Nat. Geosci. 6, 691–700 (2013).

[52] R. J. Geider, J. LaRoche, Redfield revisited: Variability of C:N:P in marine microalgae and its biochemical basis. Eur. J. Phycol. 37, 1–17 (2002).

[53] P. A. del Giorgio, J. J. Cole, Bacterial growth efficiency in natural aquatic systems. Annu. Rev. Ecol. Syst. 29, 503–541 (1998).

[54] R. LaBrie, B. Péquin, N. Fortin St-Gelais, I. Yashayaev, J. Cherrier, Y. Gélinas, F. Guillemette, D. C. Podgorski, R. G. M. Spencer, L. Tremblay, R. Maranger, Deep ocean microbial communities produce more stable dissolved organic matter through the succession of rare prokaryotes. Sci. Adv. 8, eabn0035 (2022). 35857452 10.1126/sciadv.abn0035 PMC11323801

[55] H. Teeling, B. M. Fuchs, D. Becher, C. Klockow, A. Gardebrecht, C. M. Bennke, M. Kassabgy, S. Huang, A. J. Mann, J. Waldmann, M. Weber, A. Klindworth, A. Otto, J. Lange, J. Bernhardt, C. Reinsch, M. Hecker, J. Peplies, F. D. Bockelmann, U. Callies, G. Gerdts, A. Wichels, K. H. Wiltshire, F. O. Glöckner, T. Schweder, R. Amann, Substrate-controlled succession of marine bacterioplankton populations induced by a phytoplankton bloom. Science 336, 608–611 (2012). 22556258 10.1126/science.1218344

[56] K. Krüger, M. Chafee, T. B. Francis, T. Glavina del Rio, D. Becher, T. Schweder, R. I. Amann, H. Teeling, In marine Bacteroidetes the bulk of glycan degradation during algae blooms is mediated by few clades using a restricted set of genes. ISME J. 13, 2800–2816 (2019). 31316134 10.1038/s41396-019-0476-y PMC6794258

[57] S. Vidal-Melgosa, A. Sichert, T. B. Francis, D. Bartosik, J. Niggemann, A. Wichels, W. G. T. Willats, B. M. Fuchs, H. Teeling, D. Becher, T. Schweder, R. Amann, J.-H. Hehemann, Diatom fucan polysaccharide precipitates carbon during algal blooms. Nat. Commun. 12, 1150 (2021). 33608542 10.1038/s41467-021-21009-6 PMC7896085

[58] G. Huang, S. Vidal-Melgosa, A. Sichert, S. Becker, Y. Fang, J. Niggemann, M. H. Iversen, Y. Cao, J.-H. Hehemann, Secretion of sulfated fucans by diatoms may contribute to marine aggregate formation. Limnol. Oceanogr. 66, 3768–3782 (2021).

[59] M. Garel, P. Bonin, S. Martini, S. Guasco, M. Roumagnac, N. Bhairy, F. Armougom, C. Tamburini, Pressure-retaining sampler and high-pressure systems to study deep-sea microbes under in situ conditions. Front. Microbiol. 10, 453 (2019). 31024462 10.3389/fmicb.2019.00453 PMC6465632

[60] C. Amano, T. Reinthaler, E. Sintes, M. M. Varela, J. Stefanschitz, S. Kaneko, J. Nakano, W. Borchert, G. J. Herndl, M. Utsumi, A device for assessing microbial activity under ambient hydrostatic pressure: The in situ microbial incubator (ISMI). Limnol. Oceanogr. Methods 21, 69–81 (2023). 38505832 10.1002/lom3.10528 PMC10946486

[61] K. O. Buesseler, C. H. Lamborg, P. W. Boyd, P. J. Lam, T. W. Trull, R. R. Bidigare, J. K. B. Bishop, K. L. Casciotti, F. Dehairs, M. Elskens, M. Honda, D. M. Karl, D. A. Siegel, M. W. Silver, D. K. Steinberg, J. Valdes, B. Van Mooy, S. Wilson, Revisiting carbon flux through the ocean’s twilight zone. Science 316, 567–570 (2007). 17463282 10.1126/science.1137959

[62] H. Martin, G. A. Knauer, D. M. Karl, W. W. Broenkow, VERTEX: Carbon cycling in the northeast Pacific. Deep Sea Res. A Oceanogr. Res. Pap. 34, 267–285 (1987).

[63] Y. Shen, R. Benner, T. A. B. Broek, B. D. Walker, M. D. McCarthy, Special delivery of proteinaceous matter to deep-sea microbes. Sci. Adv. 11, eadr0736 (2025). 40106540 10.1126/sciadv.adr0736 PMC11922029

[64] P. W. Boyd, H. Claustre, M. Levy, D. A. Siegel, T. Weber, Multi-faceted particle pumps drive carbon sequestration in the ocean. Nature 568, 327–335 (2019). 30996317 10.1038/s41586-019-1098-2

[65] R. R. L. Guillard, P. E. Hargraves, *Stichochrysis immobilis* is a diatom not a chrysophyte. Phycologia 32, 234–236 (1993).

[66] P. Stief, A. Kamp, B. Thamdrup, R. N. Glud, Anaerobic nitrogen turnover by sinking diatom aggregates at varying ambient oxygen levels. Front. Microbiol. 7, 98 (2016). 26903977 10.3389/fmicb.2016.00098 PMC4742529

[67] P. Stief, C. Schauberger, M. B. Lund, A. Greve, R. M. M. Abed, M. A. A. Al-Najjar, K. Attard, S. Bonaglia, J. S. Deutzmann, B. Franco-Cisterna, E. García-Robledo, M. Holtappels, U. John, A. Maciute, M. J. Magee, R. Pors, T. Santl-Temkiv, A. Scherwass, D. S. Sevilgen, D. de Beer, R. N. Glud, A. Schramm, A. Kamp, Intracellular nitrate storage by diatoms can be an important nitrogen pool in freshwater and marine ecosystems. Commun. Earth Environ. 3, 154 (2022).

[68] B. Franco-Cisterna, P. Stief, R. N. Glud, Temperature effects on carbon mineralization of sinking copepod carcasses. Mar. Ecol. Prog. Ser. 679, 31–45 (2021).

[69] U. J. Wünsch, K. R. Murphy, C. A. Stedmon, Fluorescence quantum yields of natural organic matter and organic compounds: Implications for the fluorescence-based interpretation of organic matter composition. Front. Mar. Sci. 2, 98 (2015).

[70] D. N. Kothawala, K. R. Murphy, C. A. Stedmon, G. A. Weyhenmeyer, L. J. Tranvik, Inner filter correction of dissolved organic matter fluorescence. Limnol. Oceanogr. Methods 11, 616–630 (2013).

[71] P. G. Coble, Characterization of marine and terrestrial DOM in seawater using excitation-emission matrix spectroscopy. Mar. Chem. 51, 325–346 (1996).

[72] C. H. Lochmueller, S. S. Saavedra, Conformational changes in a soil fulvic acid measured by time-dependent fluorescence depolarization. Anal. Chem. 58, 1978–1981 (1986).

[73] H. Rausch, Analysis of proteins using electrophoresis techniques. J. Protein Stud. 15, 245–258 (1981).

[74] O. H. Lowry, N. J. Rosebrough, A. L. Farr, R. J. Randall, Protein measurement with the Folin phenol reagent. J. Biol. Chem. 193, 265–275 (1951). 14907713

[75] S. Becker, A. Scheffel, M. F. Polz, J.-H. Hehemann, Accurate quantification of laminarin in marine organic matter with enzymes from marine microbes. Appl. Environ. Microbiol. 83, e03389–e03316 (2017). 28213541 10.1128/AEM.03389-16 PMC5394322

[76] M. Lever, A new reaction for colorimetric determination of carbohydrates. Anal. Biochem. 47, 273–279 (1972). 5031119 10.1016/0003-2697(72)90301-6

[77] S. Becker, J.-H. Hehemann, Laminarin quantification in microalgae with enzymes from marine microbes. Bio Protoc. 8, e2666 (2018). 10.21769/BioProtoc.2666 PMC8275226 34286020

[78] F. Franchini, M. Steinke, “Protocols for the quantification of dimethyl sulfide (DMS) and other volatile organic compounds in aquatic environments,” in *Hydrocarbon and Lipid Microbiology Protocols.* T. J. McGenity, K. N. Timmis, B. Nogales, Eds. (Springer, 2017), pp. 161–177.

[79] S. T. Chambers, C. M. Kunin, D. Miller, A. Hamada, Dimethylthetin can substitute for glycine betaine as an osmoprotectant molecule for *Escherichia coli*. J. Bacteriol. 169, 4845–4847 (1987). 3308858 10.1128/jb.169.10.4845-4847.1987 PMC213866

[80] T. Dittmar, B. Koch, N. Hertkorn, G. Kattner, A simple and efficient method for the solid-phase extraction of dissolved organic matter (SPE-DOM) from seawater. Limnol. Oceanogr. Methods 6, 230–235 (2008).

[81] J. Merder, J. A. Freund, U. Feudel, C. T. Hansen, J. A. Hawkes, B. Jacob, K. Klaproth, J. Niggemann, B. E. Noriega-Ortega, H. Osterholz, P. E. Rossel, M. Seidel, G. Singer, A. Stubbins, H. Waska, T. Dittmar, ICBM-OCEAN: Processing ultrahigh-resolution mass spectrometry data of complex molecular mixtures. Anal. Chem. 92, 6832–6838 (2020). 32298576 10.1021/acs.analchem.9b05659

[82] C. M. Bennke, G. Reintjes, M. Schattenhofer, A. Ellrott, J. Wulf, M. Zeder, B. M. Fuchs, Modification of a high-throughput automatic microbial cell enumeration system for shipboard analyses. Appl. Environ. Microbiol. 82, 3289–3296 (2016). 27016562 10.1128/AEM.03931-15 PMC4959242

