# Supplementary material for: Hydrostatic pressure induces strong leakage of dissolved organic matter from “marine snow” particles
Source: Sci Adv. 2026 Feb 4;12(6):eaec5677. doi: 10.1126/sciadv.aec5677 (PMC12871439; doi:10.1126/sciadv.aec5677)
Supplement: Supplementary file 1 — Figs. S1 to S5 Tables S1 and S2 [file sciadv.aec5677_sm.pdf]

Science Advances
AAAS

Supplementary Materials for

Hydrostatic pressure induces strong leakage of dissolved organic matter from "marine snow" particles

Peter Stief *et al.*

Corresponding author: Peter Stief, peterstief@biology.sdu.dk

Sci. Adv. **12**, eaec5677 (2026)
DOI: 10.1126/sciadv.aec5677

This PDF file includes:

Figs. S1 to S5
Tables S1 and S2

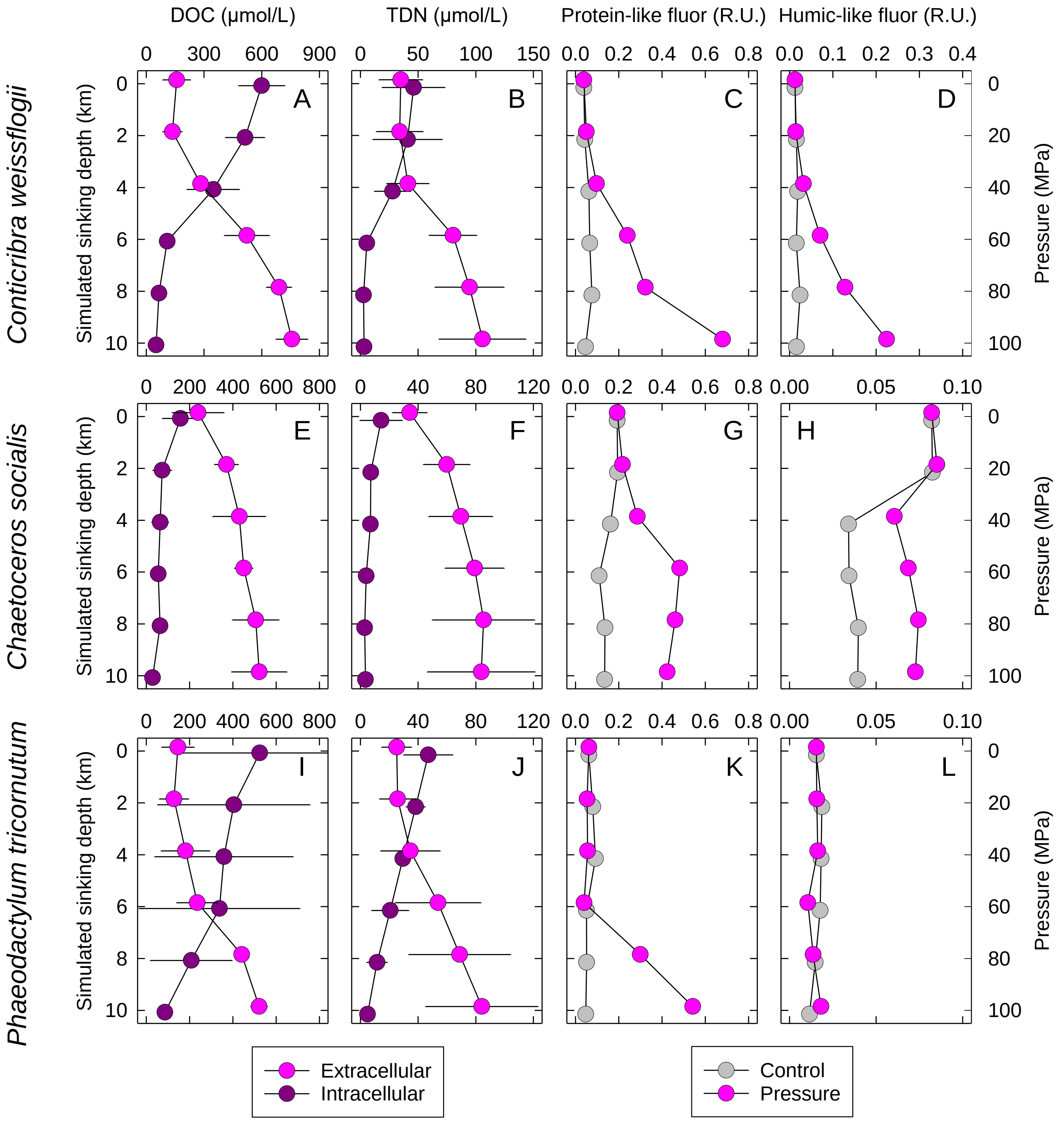

Figure S1: DOM leakage from three different diatom strains. Bulk composition and fluorescence of dissolved organic matter in incubations of cultures of three different diatom species. Hydrostatic pressure was increased by 5 MPa/d, thereby simulating the 20-day descent of the diatoms from the surface ocean (0 km ≙ 0.1 MPa) into a deep-sea trench (10 km ≙ 100 MPa) ('pressure' treatment). Pressure was kept at atmospheric level in parallel incubations ('control' treatment). Incubation temperature was 3 °C. Extra- and intracellular concentrations are expressed per sample volume and can thus be directly compared. DOC = dissolved organic carbon, TDN = total dissolved nitrogen, Fluor = fluorescence, R.U. = Raman units. Means ± standard deviation of 3 independent experiments per diatom species are shown.

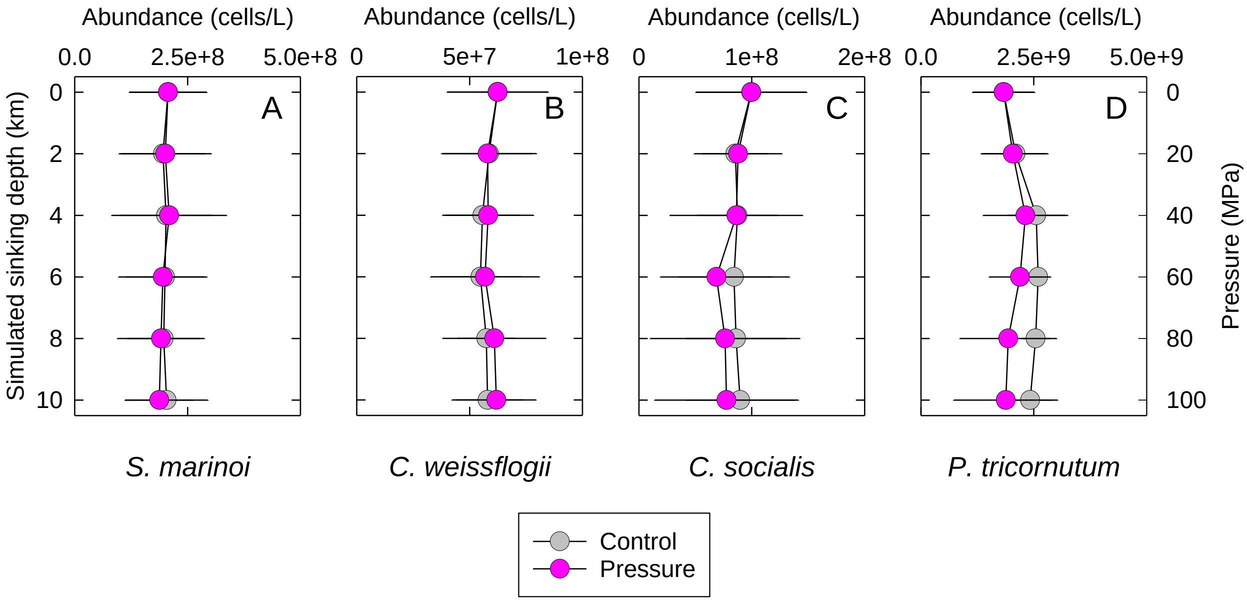

Figure S2: Cell abundance of four different diatom species. Hydrostatic pressure was increased by 5 MPa/d, thereby simulating the 20-day descent of the diatoms from the surface ocean (0 km ≙ 0.1 MPa) into a deep-sea trench (10 km ≙ 100 MPa) (‘pressure’ treatment). Pressure was kept at atmospheric level in parallel incubations (‘control’ treatment). Incubation temperature was 3 °C. Mean ± standard deviation of 3-4 independent experiments per diatom species is shown.

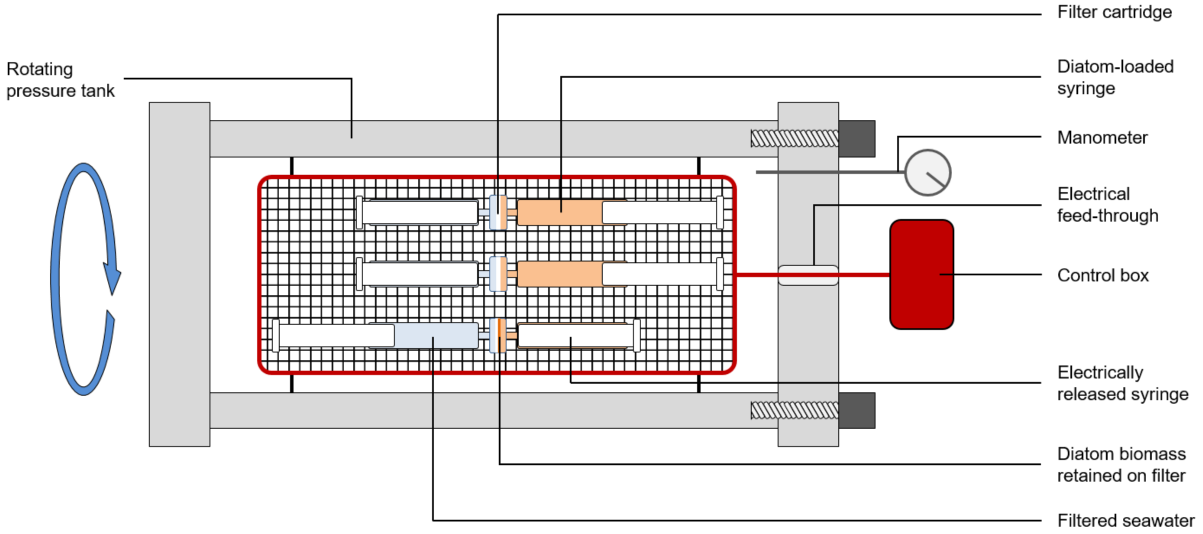

Figure S3: **Schematic of a custom-made, under-pressure filtration module.** Two 20-mL glass syringes each are tightly connected via a filter cartridge. One syringe is filled with the sample (here: diatom culture), while the other syringe remains empty. The two connected syringes are mounted onto the module with the piston of the diatom-loaded syringe fastened to a spring-operated fixture (not shown). In total, the filtration module holds three pairs of syringes and is inserted into a pressure tank equipped with an electrical feed-through in the lid. The pressure level adjusted inside the tank is transmitted into the diatom-filled syringe via the movable pistons. The three pairs of syringes can be released individually by electric signals sent through the feed-through in the lid of the pressure tank. Thereby, the contents of the diatom-filled syringe is pressed through the filter, which physically separates the diatom biomass retained on the filter from the seawater in the receiving syringe. After the filtration is completed in all three pairs of syringes (~30 s each), the tank is depressurized (~10 min), and the syringes are retrieved for sampling (~5 min). Any DOM leaking from the diatoms during pressurization is contained in the filtered seawater, whereas any DOM leaking during the subsequent depressurization does not get into contact with the filtered seawater.

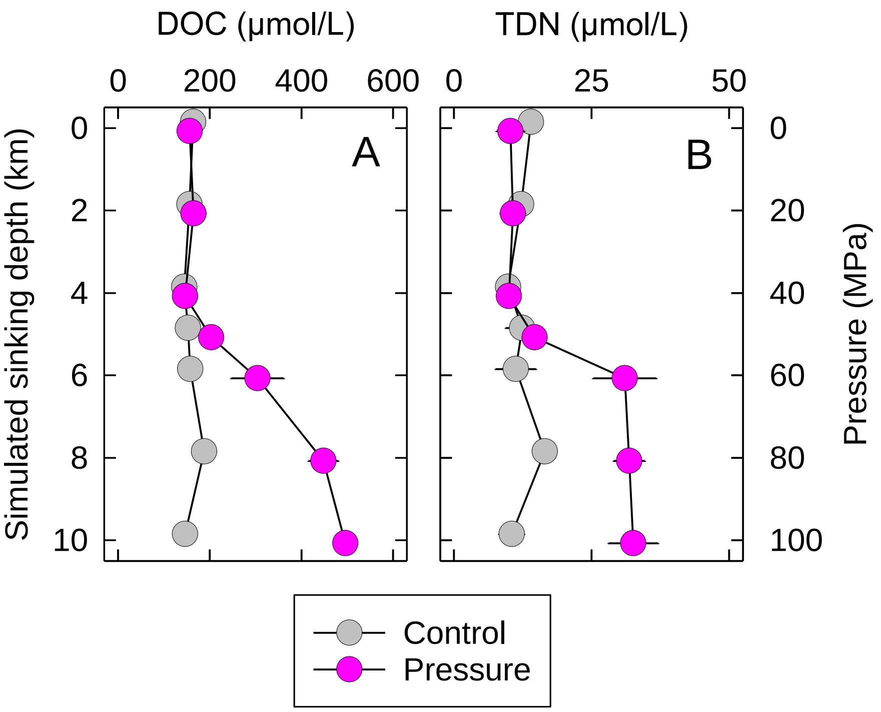

Figure S4: **Incubation of diatom cultures in the under-pressure filtration module.** In serial pressure-tank experiments, the *S. marinoi*-loaded filtration module **(Supplementary Figure S3)** was pressurized to 0.1, 20, 40, 50, 60, 80, or 100 MPa. After 30 min of pressure exposure, the three pairs of syringes were individually released and the pressure tank was depressurized. Incubation temperature was 3 °C. In the filtered seawater, (**A**) DOC and (**B**) TDN concentrations were measured. The observed patterns of DOC and TDN concentrations were consistent with results presented in **Figure 2** and **Table 1**, notably without a *prior* depressurization step. This demonstrates that pressure-induced DOM leakage occurs during pressurization rather than depressurization and is not a sample recovery artefact. Means ± standard deviation (n=3) are shown.

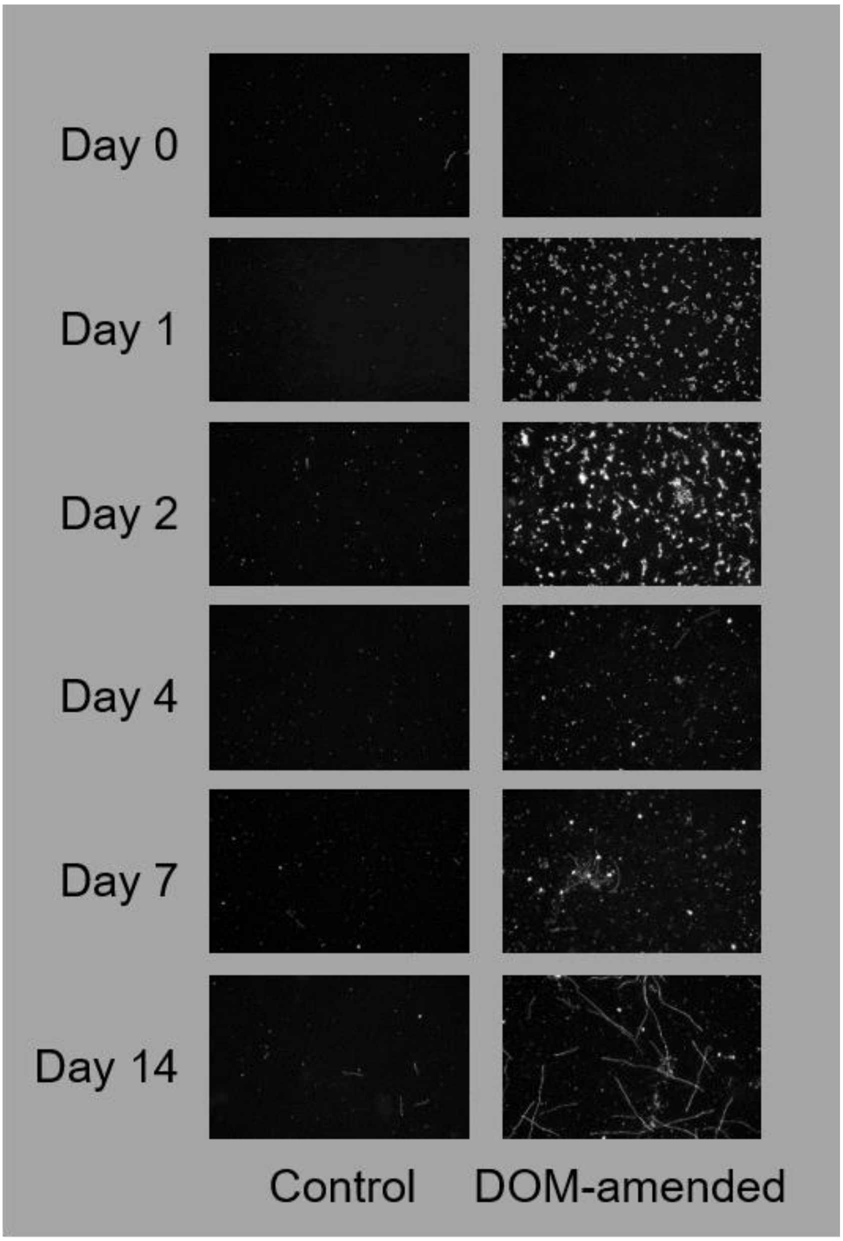

Figure S5: **Response of a natural seawater microbial community to amendment with DOM leachates of *S. marinoi*** incubated at atmospheric pressure and 15°C for two weeks. Time course of bacterial cell morphologies in DOM-amended vs. control incubations. Representative microscopic images of DAPI-stained bacterial cells are shown.

Table S1: Statistics summary for replicated data: Two-way repeated-measures ANOVA.

Fig.	Sample	Variable	Repl.	Treatment	Time	Interaction
2A	Aggregates	Extracellular DOC	21	$F_{(1,120)}$=30.155 p<0.001	$F_{(5,40)}$=5.467 p<0.001	$F_{(5,240)}$=16.527 p<0.001
2B	Aggregates	Extracellular TDN	21	$F_{(1,120)}$=10.859 p=0.003	$F_{(5,40)}$=24.259 p<0.001	$F_{(5,240)}$=7.225 p<0.001
2C	Aggregates	Protein-like fluorescence	3	$F_{(1,12)}$=320.8 p=0.003	$F_{(5,4)}$=31.203 p<0.001	$F_{(5,24)}$=74.965 p<0.001
2D	Aggregates	Humic-like fluorescence	3	$F_{(1,12)}$=6.471 **p=0.126**	$F_{(5,4)}$=7.462 p=0.004	$F_{(5,24)}$=5.361 p=0.012
2E	*S. marinoi* culture	Extracellular DOC	4	$F_{(1,18)}$=32.121 p=0.011	$F_{(5,6)}$=13.031 p<0.001	$F_{(5,36)}$=21.455 p<0.001
2F	*S. marinoi* culture	Extracellular TDN	4	$F_{(1,18)}$=124.153 p=0.002	$F_{(5,6)}$=8.549 p<0.001	$F_{(5,36)}$=29.74 p<0.001
3A	*S. marinoi* culture	Extracellular DOC	4	$F_{(1,18)}$=32.121 p=0.011	$F_{(5,6)}$=13.031 p<0.001	$F_{(5,36)}$=21.455 p<0.001
3B	*S. marinoi* culture	Extracellular proteins	4	$F_{(1,18)}$=82.534 p=0.003	$F_{(5,6)}$=3.498 p=0.027	$F_{(5,36)}$=11.256 p<0.001
3C	*S. marinoi* culture	Extracellular carbohydrates	3	$F_{(1,12)}$=7.687 **p=0.069**	$F_{(5,4)}$=7.453 p=0.001	$F_{(5,24)}$=13.794 p<0.001
3G	*S. marinoi* culture	Intracellular DOC	4	$F_{(1,18)}$=15.208 p=0.030	$F_{(5,6)}$=23.1 p<0.001	$F_{(5,36)}$=10.066 p<0.001
3H	*S. marinoi* culture	Intracellular proteins	3	$F_{(1,12)}$=3.41 **p=0.162**	$F_{(5,4)}$=2.416 **p=0.085**	$F_{(5,24)}$=3.166 p=0.038
3I	*S. marinoi* culture	Intracellular carbohydrates	3	$F_{(1,12)}$=5.115 **p=0.109**	$F_{(5,4)}$=5.589 p=0.004	$F_{(5,24)}$=10.997 p<0.001
4A	Seawater	Extracellular DOC	6	$F_{(1,30)}$=967.976 p<0.001	$F_{(5,10)}$=33.236 p<0.001	$F_{(5,60)}$=30.85 p<0.001
4B	Seawater	Extracellular TDN	6	$F_{(1,30)}$=465.922 p<0.001	$F_{(5,10)}$=5.179 p=0.002	$F_{(5,60)}$=0.744 **p=0.598**
4C	Seawater	Extracellular proteins	6	$F_{(1,30)}$=227.559 p=0.004	$F_{(5,10)}$=40.575 p<0.001	Not all time points match
4D	Seawater	Extracellular carbohydrates	6	$F_{(1,30)}$=4161 p<0.001	$F_{(5,10)}$=88.066 p<0.001	Not all time points match
4G	Seawater	Respiration rate	3	$F_{(1,44)}$=1866 p<0.001	$F_{(21,4)}$=66.599 p<0.001	$F_{(21,88)}$=46.801 p<0.001
4H	Seawater	Bacterial abundance	3	$F_{(1,12)}$=11568 p<0.001	$F_{(5,4)}$=20.091 p<0.001	$F_{(5,24)}$=53.037 p<0.001

Table S2: Elemental composition of four diatom species and maximum relative leakage as dissolved organic carbon (DOC) and total dissolved nitrogen (TDN) upon pressure exposure.

Species	Taxonomy	Carbon contents (pg/cell)	Nitrogen contents (pg/cell)	Biomass C:N ratio (molar)	Max. DOC leakage (%)	Max. TDN leakage (%)	Leachate C:N ratio (molar)
Skeletonema marinoi	Coscinodiscophyceae (centric)	38.3 ± 0.4	4.2 ± 0.1	10.63 ± 0.37	42.1 ± 9.5	63.0 ± 12.2	7.37 ± 2.15
Conticribra weissflogii	Coscinodiscophyceae (centric)	251.4 ± 11.2	14.1 ± 1.0	20.75 ± 1.74	25.7 ± 6.3	76.5 ± 26.6	7.11 ± 2.95
Chaetoceros socialis	Coscinodiscophyceae (centric)	51.2 ± 1.5	5.4 ± 0.2	11.13 ± 0.52	17.0 ± 9.5	34.1 ± 15.1	5.68 ± 3.97
Phaeodactylum tricornutum	Bacillariophyceae (pennate raphid)	13.1 ± 0.6	1.2 ± 0.1	12.48 ± 0.96	17.3 ± 1.8	42.6 ± 29.5	5.16 ± 3.47

Solute leakage per diatom cell was calculated as the difference in extracellular concentration at elevated vs. atmospheric pressure. Maximum relative leakage was observed at 60, 80, or 100 MPa within a given pressure experiment. Molar C:N ratios were calculated for aggregate and diatom biomass and for DOM leachates. To this end, the measured TDN concentrations were adjusted by –1 µmol N/L to account for the minor concentrations of dissolved inorganic nitrogen (i.e., nitrate and ammonium) measured in the incubation vials and thereby DON concentrations were estimated. Means ± standard deviation of 4 (*S. marinoi*) or 3 (all other species) independent pressure experiments are shown.
